# The majority of genes in the pathogenic *Neisseria *species are present in non-pathogenic *Neisseria lactamica*, including those designated as 'virulence genes'

**DOI:** 10.1186/1471-2164-7-128

**Published:** 2006-05-30

**Authors:** Lori AS Snyder, Nigel J Saunders

**Affiliations:** 1 Bacterial Pathogenesis and Functional Genomics Group, Sir William Dunn School of Pathology, University of Oxford, Oxford, OX1 3RE, UK

## Abstract

**Background:**

*Neisseria meningitidis *causes the life-threatening diseases meningococcal meningitis and meningococcal septicemia. *Neisseria gonorrhoeae *is closely related to the meningococcus, but is the cause of the very different infection, gonorrhea. A number of genes have been implicated in the virulence of these related yet distinct pathogens, but the genes that define and differentiate the species and their behaviours have not been established. Further, a related species, *Neisseria lactamica *is not associated with either type of infection in normally healthy people, and lives as a harmless commensal. We have determined which of the genes so far identified in the genome sequences of the pathogens are also present in this non-pathogenic related species.

**Results:**

Thirteen unrelated strains of *N. lactamica *were investigated using comparative genome hybridization to the pan-*Neisseria *microarray-v2, which contains 2845 unique gene probes. The presence of 127 'virulence genes' was specifically addressed; of these 85 are present in *N. lactamica*. Of the remaining 42 'virulence genes' only 11 are present in all four of the sequenced pathogenic *Neisseria*.

**Conclusion:**

Assessment of the complete dataset revealed that the vast majority of genes present in the pathogens are also present in *N. lactamica*. Of the 1,473 probes to genes shared by all four pathogenic genome sequences, 1,373 hybridize to *N. lactamica*. These shared genes cannot include genes that are necessary and sufficient for the virulence of the pathogens, since *N. lactamica *does not share this behaviour. This provides an essential context for the interpretation of gene complement studies of the pathogens.

## Background

*Neisseria meningitidis *and *Neisseria gonorrhoeae *are closely related bacterial pathogens, which share many common genes with a high degree of sequence identity (typically greater than 95%). DNA relatedness studies cluster the commensal *N. lactamica *with the two pathogenic *Neisseria *spp. [1]. Sequence identity of housekeeping genes also clusters *N. lactamica *with the pathogens [2]. Unlike *N. meningitidis *and *N. gonorrhoeae*, the rare cases of disease caused by *N. lactamica *are largely due to some compromise in the patient, and it is not normally pathogenic [3-5].

The pan-*Neisseria *microarray-v2 was designed so that common probes are used to address all coding regions thus far identified in the pathogenic *Neisseria *spp., with 2716 probes to all of the probable coding regions from the four complete neisserial genomes, plus 129 probes for additional neisserial genes [6]. The probes were designed, based upon a comparative genomic analysis, to the most conserved regions within the genes, to optimize the microarray for use in investigations of strains against which it was not designed. Because the probes are minimally genome specific, being within the most conserved areas of the genes, it is possible to also use the pan-*Neisseria *microarray-v2 to assess the gene complement of the related non-pathogenic species *N. lactamica*.

The range of tools available to address the similarities, differences, and evolutionary relationships between bacterial species has been extended with the availability of multiple genome sequences from within related species groups and the development of microarray-based comparative genome hybridization studies. While traditional methods of comparison and typing are based upon the comparison of divergence within genes that are common and highly conserved, the genomic approach facilitates a very different and complementary method of investigation that allows the commonality and flux of genes, and combinations of genes, within and between species to be addressed. Methods such as MLEE and MLST broadly address genes that are considered to be essential and under minimal functional selection, and are thus under sufficiently low selective pressure to show changes over a useful period of evolutionary time for the purposes of a particular study. It is also the case, however, that horizontal exchange of genes and gene-systems is occurring that directly affect behaviour and these are frequently directly under selection. In two recent studies of another relatively panmictic species, *Helicobacter pylori*, findings relevant to this have recently been made. Firstly, it was shown that rather than being relatively immune to recombination, exchange is polarized in both "highly conserved" genes associated with central metabolic processes as well as those associated with strain-dependent behaviour. The former is driven by homology-dependent recombination and their highly conserved nature, and the latter by functional selection [7]. Secondly, it has been demonstrated that while differences in genes under selection broadly associate with the MLST-based population structure, clear examples of exchange occur between what the traditional relatedness trees consider to be very remote unrelated strains (for example between the hpAfrica2 population and hpAfrica1) (Salaün & Saunders – unpublished data). This highlights the need for, and the complementary nature of, comparative genome hybridization studies to determine gene complements in studies of population structures and their evolution. This is because this approach measures a different comparatively slow type of change (the acquisition and loss of whole genes) that is potentially directly related to behavior. This may be key to determining which changes underlie the emergence and relative success of different clonal clusters, especially within relatively panmictic populations. In the context of studies of bacterial pathogens this approach has the potential to identify those genes or gene combinations that are responsible for the periodic increases and decreases in strains with differing pathogenic potentials.

Thirteen unrelated strains of *N. lactamica *were assessed through comparative genome hybridization to the pan-*Neisseria *microarray-v2 to determine which genes thought to be associated with virulence traits of the pathogenic *Neisseria *spp. are present in this non-pathogenic species. The pan-*Neisseria *microarray-v2 contains probes for the genes from the genome sequences of *N. gonorrhoeae *strain FA1090, *N. meningitidis *strain MC58, *N. meningitidis *strain Z2491, *N. meningitidis *strain FAM18, and to the genes from the *N. gonorrhoeae *strain MS11 Gonococcal Genetic Island, neisserial genes from GenBank/EMBL that did not yet have probes, and newly identified genes from Minimal Mobile Elements (see Additional files 2 &3). While previously used subtractive methods [8] have been able to identify some of the genes missing from a small number of *N. lactamica *strains, hybridization to the microarray probes provides positive identification of the sequences that are present in this species.

## Results

### Hybridization of *N. lactamica *to the pan-*Neisseria *microarray-v2

**Figure 1:**
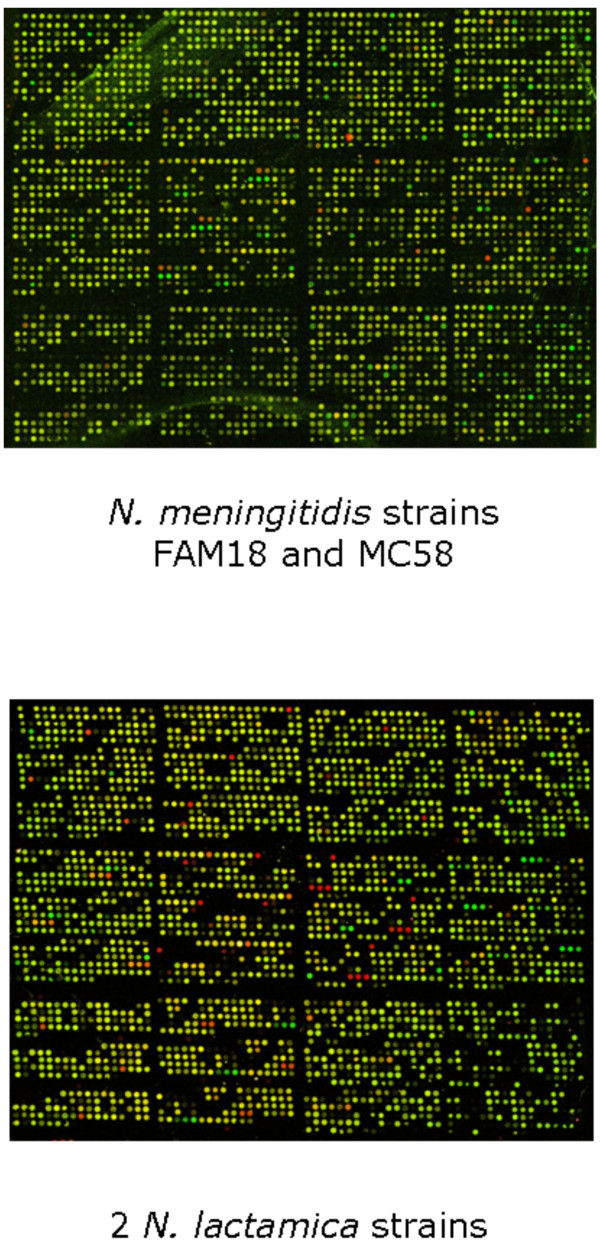
Hybridization to the pan-*Neisseria *microarray-v2.

The hybridization of the *N. lactamica *strains (Table 1) to a large proportion of the probes of the pan-*Neisseria *microarray-v2 is both a validation of the design strategy used for the microarray (see Additional file 3) and highlights the similarity of these species. This data can be easily visualized in its genomic context at , where GBrowse databases detail the *N. gonorrhoeae *strain FA1090 and *N. meningitidis *strain MC58 and Z2491 genome sequences, their annotations, microarray probe locations and sequences, and indicate those probes that hybridize with *N. lactamica *in this study. In general, with *N. lactamica *neither the intensity of the hybridization signals nor the percentage of the microarray probes hybridized significantly differs from that seen in similar *N. meningitidis *or *N. gonorrhoeae *comparative genome hybridizations (Figure 1).

**Table 1:** Strains used in this study.

Species	Strain	ST by MLST
*N. lactamica*	10102M	ST-613
*N. lactamica*	14804	ST-595
*N. lactamica*	08802S1	ST-640
*N. lactamica*	908	ST-598
*N. lactamica*	4116	ST-615
*N. lactamica*	1520	ST-592
*N. lactamica*	5804	ST-616
*N. lactamica*	11004	ST-1202
*N. lactamica*	224	ST-624
*N. lactamica*	310	ST-643
*N. lactamica*	12024	ST-1205
*N. lactamica*	2510	ST-609
*N. lactamica*	Stephens	ND

### *N. lactamica *hybridizes to 'virulence gene' probes

**Figure 2:**
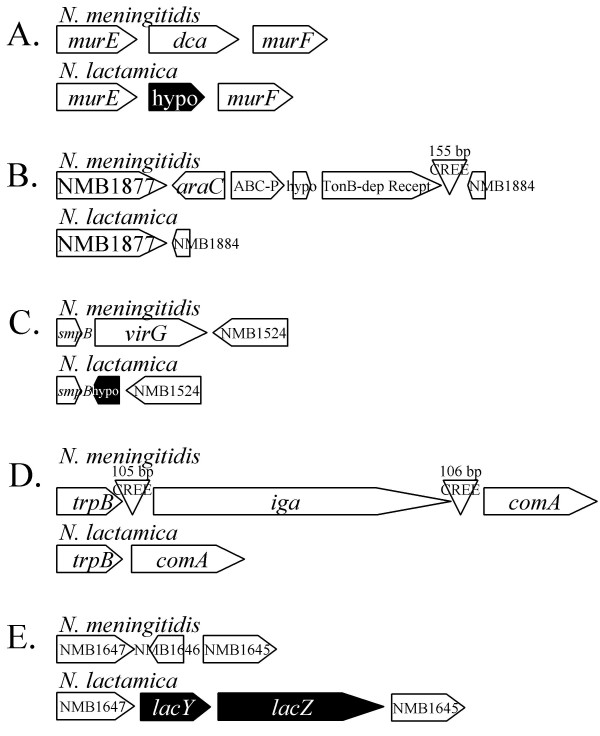
The chromosomal locations of the 6 'virulence genes' present in all pathogenic *Neisseria *spp. genome sequences, but absent from the *N. lactamica *genome sequence and for which no *N. lactamica *strains hybridized to the pan-*Neisseria *microarray-v2. Panel A: the region between *murE *and *murF *contains *dca *in the pathogen genome sequences and a hypothetical gene in the *N. lactamica *genome sequence. Panel B: the region between NMB1877 and NMB1884 contains the 'virlence genes' NMB1880 (ABC-P, ABC transport periplasmic substrate-binding protein) and NMB1882 (TonB-dependent receptor) as well as a hypothetical gene (NMB1881) and an *araC*-like regulator in the pathogen genome sequences, which are all absent in this location in the *N. lactamica *genome sequence. The probe for NMB1878, the *araC *regulator hybridized to all *N. lactamica *strains assessed by CGH; this may be due to another *araC*-like regulator in another location or the presence of this regulator in this location in all the assessed strains. Panel C: the region between *smpB *and NMB1524 contains *virG *in the pathogen genome sequences and a hypothetical gene in the *N. lactamica *genome sequence. Panel D: the region between *trpB *and *comA *contains *iga*, flanked by Correia Repeat Enclosed Elements (CREE) commonly found in the neisserial genomes; *iga *and the CREEs are absent in the *N. lactamica *genome sequence. Panel E: the region between NMB1647 and NMB1645 contains a putative hemolysin encoded by NMB1646 in the pathogenic genome sequences, while in the *N. lactamica *genome sequence this is the location of *lacY *and *lacZ*, which confer the characteristic lactose fermentation phenotype of *N. lactamica*.

A set of genes identified in the literature [8-10] to be involved in the virulence or pathogenesis of the *Neisseria *spp. (see Additional file 1) were specifically assessed through manual interpretation of the microarray image data. The microarray scan images of each spot feature were individually analyzed to determine hybridization to the probes for this subset of genes. Most of these genes were identified in *N. lactamica *(Table 2). Of the 127 virulence-associated genes assessed (of which 83 are present in all of the 4 pathogen genome sequences), 85 were present in at least one of the strains of *N. lactamica*. Many of the 42 remaining pathogen-specific genes are not present in all of the pathogenic *Neisseria *spp. genome sequences. Based upon microarray hybridizations, 11 of these 'virulence-associated' genes that are universally present in the pathogen sequences did not produce hybridization signals from any of the *N. lactamica *strains tested (Table 3). Of these, analysis of the currently unpublished *N. lactamica *genome sequence of another strain of this species (The Wellcome Trust Sanger Institute – unpublished) indicates that divergent homologues of the pilus-associated genes *pilC*, *pilD*, *pglD*, and *pglF *are present in this sequenced strain of *N. lactamica*, as is a divergent version of the antigen-encoding *ssa*1 gene. For the remaining 6 genes, the equivalent genomic region could be found in each case, yet these genes were absent, both here (Figure 2) and in the genome sequence as a whole. In the cases of *dca*, *virG*, and the NMB1646 hemolysin gene, alternative genes are present in these locations (Figure 2, panels A, C, and E). These results suggest that of the total number of currently recognized 'virulence genes', excluding the influence of allelic differences, the presence of only six can now be considered to be pathogen-specific. Only two of these six genes, *iga *and *dca*, have previously been reported to be pathogen-specific [4,8,9,11-14].

**Table 2:** Comparative genome hybridization to 'virulence gene' probes.

Genes present in all of the strains of *N. lactamica *tested	Genes present in some of the strains of *N. lactamica *tested
Surface proteins	Surface proteins
*opa*, *tspA*, *envA*, *rmpM*, *ompH*, *porB*, NMB1946, *mtrR*, *mtrD*, *mtrE*, *hsf*, *nlpD*	*tspB*, *mtrC*, *omp*85, *hap*
Iron acquisition	Iron acquisition
*tbp*2, *fbpA*, *bfrA*, *hemH*, NMB1989–NMB1991	*lbpA*, *lbpB*, *bfrB*, *bcp*, *fbpB*, *tbp*1, NMB2132
Pilus associated	Pilus associated
* pglE*, *pilG*, *pilF*, *pglH*	* pilE*, *pilT*, NMB0051, *pglA*/*pgtA*, *pglB*, *pglC*, *pglG*, *pglI*/*wbpC*
LPS biosynthesis/regulation	LPS biosynthesis/regulation
*lgtA*, *lgtB*, *lgtC*, *lgtD*, *lgtG*, *rfaC*, *rfaE*, *lpxB*, *lpxD*, *fabZ*, *pgm*, *phoQ*/*misS*	*rfaD*, *rfaF*, *rfaK*, *lgtF*, *lpxA*, *kdtA*, *phoP*/*misR*,
Others	Others
* prc*, *hecB*, hemagglutinin/hemolysins NMB0493 & NMB1214, endonuclease NMB0533, VapD-related NMB1753, macrophage infectivity potentiator NMB1567, sialyltransferase NMB0922, adhesion NMB0586, TonB-dependent receptor NMB1449, toxin-activating NMB1763	*kat*, *dsbA*, *sodB*, *norZ*, *vapA*, *fur*, *vacJ*, hemagglutinin/hemolysin NMB1768, protease NMB2127, macrophage infectivity potentiator NMB0995, toxin-activating NMB1210, TonB-dependent receptors NMB1346 & NMB1829

**Table 3:** Pathogen-specific virulence genes absent in *N. lactamica*.

**No hybridization on microarray^a^**	***N. lactamica *genome sequence results^b^**	**Final results**
*ssa*1	Present, but divergent	Present
*pilC*	Present, but divergent	Present
*pilD*	Present, possibly divergent in strains tested	Present
*pglD*	Present, but divergent	Present
*pglF*	Present, possibly divergent in strains tested	Present
*dca*/*pptA*	Absent. The equivalent region contains an alternative hypothetical gene.	Absent
ABC transport (NMB1880)	Absent in genome sequence	Absent
TonB-dependent receptor, NMB1882	Absent in genome sequence	Absent
*virG*	Absent. The equivalent region contains an alternative hypothetical gene.	Absent
*iga *encoding IgA protease	Absent in genome sequence	Absent
hemolysin, NMB1646	Absent. The equivalent region contains alternative gene sequences.	Absent

^a^Virulence-associated genes that are present in all strains of the pathogenic *Neisseria *spp., but did not produce positive microarray hybridizations with *N. lactamica*.

^b^Approximately half of these genes are present in the unpublished genome sequence of *N. lactamica*, where sequence divergence from the pathogenic sequence can be observed.

### *N. lactamica *contains most of the genes in the pathogenic *Neisseria *spp., including some previously reported as pathogen-specific

When the complete microarray dataset was interrogated, 2,137 probes hybridized to at least one of the strains of *N. lactamica *(see Additional file 2). Of these, 46 are strain specific, with the majority of these (33) being in the strain that was not part of the MLST set. The previously reported study of relatedness [1] of the neisserial species is probably heavily influenced by the genes that are shared between *N. lactamica *and the pathogenic *Neisseria *spp.

The *N. lactamica *strains used in this study possess several genes previously reported as pathogen-specific, including *lipA*, *bioA*, *secY *[9], *mafA *[15], and *dsbA *[9,15]. Of the 2,845 probes on the pan-*Neisseria *microarray-v2, 1,473 will hybridize to orthologues present in all four of the pathogenic sequenced strains, and the remainder addresses strain- or species- specific genes or highly divergent alleles. Of the 1,473 probes to genes that are universally present in the four pathogens' genome sequences, 1,373 hybridize with the *N. lactamica *strains tested.

### Comparison of our findings with those reported using the BμG@S *Neisseria *microarray

Following the design and public availability of the original pan-*Neisseria *microarray-v1 in 2002 (available from Prof. John Davies, Monash University, Australia) [16], the Bacterial Microarray Group at St. George's (BμG@S) designed a second, independent multi-strain *Neisseria *microarray that became available in 2004. During the time in which publication of this study was being pursued, the Bacterial Microarray Group published a study of the hybridization of several different *Neisseria *spp. to their microarray. Readers of other reports of neisserial microarray-based studies should note that the investigators at BμG@S have also chosen to call their microarray the pan-*Neisseria *microarray, but this is not the same microarray used in our study. In their study, Stabler *et al *[17] hybridized two *N. lactamica *strains (L13 and L18) to their microarray. We have previously used these strains for other studies [18-20], but they were not included in the current study because they do not currently form part of a collection of known diversity and on their own do not represent a sufficient sample to address our experimental question related to *N. lactamica*. We had instead chosen to evaluate the gene complements of 12 MLST analyzed strains to assess the genes present in *N. lactamica*. To this data we added one other *N. lactamica *strain that we had used previously [11,19]. In addition, our study sought to identify genes that are present in *N. lactamica*, not those that are absent from the commensal species, as was the goal of the Stabler *et al *[17] study.

Several of the genes reported by Stabler *et al *[17] as being absent from the commensal *Neisseria *spp., including their two strains of *N. lactamica*, are present in our *N. lactamica *hybridizations (Table 4). Whether these are due to differences in strain gene complement, microarray design, or data interpretation cannot be determined. In cases where the gene is present in only a few of our *N. lactamica *strains (see Additional file 2), the former may be the case, but where the gene probe hybridization is present in the majority of our strains, the later two points must be considered.

**Table 4:** Genes reported as "absent from all commensal *Neisseria *species" by Stabler *et al*., 2005, which were positively hybridized in our commensal collection.

Positive hybridizations with our *N. lactamica *collection:	
NMB0239	
NMB0240	
NMB0294	
NMB0389	
NMB0832	
NMB1719	

Positive hybridizations in other commensal species:	

NMB0083	*Neisseria elongata, Neisseria flavescens*
NMB0084	*Neisseria flavescens*
NMB0226	*Neisseria sicca*
NMB0227	*Neisseria sicca*
NMB0229	*Neisseria sicca*
NMB0293	*Neisseria elongata, Neisseria macacae, Neisseria sicca, Neisseria subflava*
NMB0431	*Neisseria flavescens, Neisseria macacae, Neisseria sicca, Neisseria subflava*
NMB0435	*Neisseria flavescens, Neisseria macacae, Neisseria sicca, Neisseria subflava*
NMB0473	*Neisseria flavescens*
NMB0474	*Neisseria flavescens*
NMB0486*	*Neisseria sicca*
NMB0654	*Neisseria polysaccharea, Neisseria sicca*
NMB1400	*Neisseria mucosa*
IS30	*Neisseria elongata, Neisseria macacae, Neisseria sicca, Neisseria subflava*

* 100% identical sequences are annotated as NMA1167, NMA1999, NMB0970, and NMB1741.

For purposes of comparing their findings with ours, the reported *pilE *and associated silent *pilS *cassettes were not evaluated, since the possession of type II pili by *N. lactamica *[21] and pathogenic *Neisseria *strains such as *N. meningitidis *strain FAM18 [22] are well known and cannot be considered a differentiating factor between the pathogenic and commensal *Neisseria *spp. In addition, the results presented in Table 3 of absent genes in Stabler *et al *[17] includes separately listed copies of identical genes: NMA1167 and NMA1999 (100% DNA ID; NMB0486/NMB0970/NMB1741); and 9 reported copies of IS30. Discounting the additional copies of genes and the *pilE/pilS *sequences, 38 genes are reported to be absent in the commensal *Neisseria *spp. by Stabler *et al *[17]; in contrast, we find that 6 of these are present in our hybridizations with *N. lactamica*, and a further 14 produced positive hybridizations to our pan-*Neisseria *microarray-v2 with other commensal species (Table 4). This highlights the problematic nature of reporting an absence based on a lack of hybridization in microarray and similar hybridization-based studies, which is why our study was focused in the genes present in *N. lactamica*.

Of the remaining 18 genes reported by Stabler *et al*. that also did not give positive hybridization results in our study, four are within Minimal Mobile Elements (MMEs; NMB0415, NMB0468, NMB0470, and NMB1621), one (NMA0368) is lost upon the acquisition of XNG1985 to XNG1898, eight (NMB0230, NMB0304, NMB1737, NMB1738, NMA0510, NMA1218, NMA1219, and NMA1221) are associated with IS elements, and one is IS150. This is consistent with these being genuinely strain and species differentiating genes based upon other evidence, reflecting their strain-specific and mobile nature. In summary, of the 38 genes reported as absent in the commensals by Stabler *et al*., 20 are present in our hybridizations and 14 are probably genuine differences, but are associated with strain-specific mobile elements.

It is interesting to note that of the 6 pathogen-specific genes identified in our study (Table 3), *iga*, NMB1880, NMB1882, *virG*, and NMB1646 are not reported as absent by Stabler *et al *[17], however their absence in our hybridizations is consistent with their absence in the *N. lactamica *genome sequence (Figure 2). The apparent presence of IgA protease in their commensal *Neisseria *collection is particularly of note, however this is not discussed in their manuscript. In light of the previously established absence of IgA protease from *N. lactamica *[13], this observation must be assumed to be false positive data, either due to cross-hybridization or data interpretation issues.

## Discussion

In this study we were able to determine that 2,137 out of the 2,845 pan-*Neisseria *microarray-v2 probes hybridize in at least one strain of *N. lactamica*, that 85 of the 127 'virulence genes' are present in this non-pathogen, and that 1,373 of the 1,473 probes (93%) to genes common to all of the four pathogenic *Neisseria *spp. genomes are present. The genomic locations of the hybridized probes can be visualized at , which presents this data in its genomic context relative to each of the published genome sequences. For the gonococcal database it also clearly relates the annotation used in this study, to that in GenBank (AE004969). The threshold for the interpretation of the microarray data was deliberately set to be highly stringent, so that a positive indication of gene presence could be reliably interpreted even in the context of the extension of this microarray to use on a species against which it was not designed. The data was then pooled, so that assay-to-assay variation in gene presence, hybridization, and divergence should not contribute to the overall gene presence results for the species.

Although probes on the pan-*Neisseria *microarray-v2 are designed to the most conserved regions of the genes from the pathogenic *Neisseria *spp., where multiple sequences of individual genes are available, substantially divergent sequences will not hybridize to the probes on the microarray. There can be differences (probably greater than at least 20% divergence) between different allelic versions of genes that would lead to the lack of hybridization to the probes. Indeed, there are relatively weak hybridizations to some of the probes for genes that are known to have multiple divergent alleles or undergo antigenic variation, most notably to the various *opa *probes. The Sanger Institute's annotation and publication of the complete genome of a different strain of *N. lactamica *may reveal that some particularly divergent gene families that appear to be absent by microarray hybridization are actually present in *N. lactamica*, but are too divergent to be detected by our microarray probes (as in Table 3). It is therefore likely that some of the probes that did not hybridize will be revealed to be due to sequence divergence or other limitations of microarray methodology. However, such divergence is likely to be associated with functional differences between the encoded proteins. Likewise, once complete, the genome sequence of *N. lactamica *will identify genes that are not in the pathogenic *Neisseria *spp. strains studied to date and are not probed by the pan-*Neisseria *microarray-v2, such as the *N. lactamica *homologue of the *Haemophilus influenzae licA*, which is responsible for the presence of phosphorylcholine on the LPS of these commensal bacteria [23]. Such genome-wide analysis of the unpublished *N. lactamica *data is beyond the permissions given by the Sanger Institute for the use of their data before publication, therefore a genome-wide comparison of these results with the *N. lactamica *genome sequence has not been performed.

Due to the nature of microarray hybridizations, which are much like the interpretations of Southern blot data [24], only a positive hybridization can reliably be interpreted. The lack of hybridization to a probe spot is not interpretable data, since the negative result can arise from a number of factors that are not necessarily related to the absence of the gene. As can be seen in Table 3, the probes may not hybridize, although the gene sequence may be present. From a sequence standpoint, this can be due to divergence of the gene or absence of a portion of the gene that includes the gene probe. From a technology standpoint, some probes never function properly, due to secondary structures formed by the probe on the slide surface or the target in solution that prevent binding. Also, signal can be lost due to too little probe material, failure of the probe to transfer to the slide surface, pin failure in printing, variations in the slide surface, and variations in target labeling using random primers. Therefore, it is not possible to robustly interpret the lack of hybridization to the probes as a definitive indication of gene absence, which is why this study has focused on the identification of the genes present (rather than absent) in *N. lactamica*. If a reader so wishes, absent genes and regions can be easily inferred from the GBrowse databases, but the nature of this data should be carefully considered. Comparison of our data to the report of gene absence from the microarray hybridizations of Stabler *et al *[17] highlights the problematic nature of reporting negative hybridization results. Additionally, the genes with positive hybridizations indicate only that the sequence is present and cannot rule out frame-shifts or other inactivating mutations of these genes, which may become apparent from the *N. lactamica *genome sequence upon its completion. An investigation of the regions specific to the pathogens has already been conducted [8], which this study complements.

The behaviors of *N. gonorrhoeae*, *N. meningitidis*, and *N. lactamica *are distinct and clearly differentiate these three species. In other bacterial species, the differentiation between pathogenic and non-pathogenic related bacteria has frequently been associated with the acquisition/presence of key 'virulence genes'. For example, the CTX prophage [25] and VPI [26] in *Vibrio cholera*, which encode cholera-toxin and toxin coregulated pilus; the LEE [27] in *E. coli *responsible for the attaching and effacing effects in EPEC and EHEC strains; and the species-defining SPI1 and SPI2 of *Salmonella *[28,29], which encode type III secretion systems. In the *Neisseria *spp., however, no such clear-cut event in the evolutionary history of these organisms has been identified to-date to account for the behavioural differences between these species, although the presence of the capsule locus is usually associated with disease-causing isolates of *N. meningitidis*. From the results presented here, it would appear that the events which lead to the evolution of the behaviors of the three closely related *N. gonorrhoeae*, *N. meningitidis*, and *N. lactamica *species have been far more subtle than in other pathogenic species groups, and that the basis for their behavioral differences may not be defined by a single event, a single set of genes, or loss of a single function. Instead, a complex integrated network of genes, regulation, and diversity of function in common genes may be responsible. For example, there are links between the ability to phase vary the *pgtA *gene in *N. gonorrhoeae *and the organism's ability to cause disseminated disease [30]. Thus, two strains of *N. gonorrhoeae *may be nearly identical, but due to a few polymorphisms in the repeat tract of *pgtA *they may have different disease potentials. As an example of differential regulation between the pathogenic species, the expression of the Mtr efflux pump, which is important for *N. gonorrhoeae *survival in the female genital tract [31], is not regulated by MtrR in *N. meningitidis *[32]. In addition, we have found that the MarR regulator controls different genes in the two pathogenic *Neisseria *spp. (Snyder & Saunders – unpublished data). The genetic similarities and differences between the pathogens and *N. lactamica *identified in this study provide an additional framework from which to pursue these issues.

From a practical perspective, because natural immunity can follow colonization with *N. lactamica*, it can be argued that an ideal vaccine would protect against both pathogenic species but not against *N. lactamica*. However, this study shows that the number of such candidates is very limited. Modeling of meningococcal and *N. lactamica *carriage shows that colonization with the commensal provides some immunological protection against the pathogen. Vaccination against something common to *N. lactamica *and the pathogens that reduces the carriage of *N. lactamica *is predicted to negatively impact the effectiveness of immunization programs [33]. This study shows that there may be as few as 100 potential candidates for such a vaccine (1,373 of 1,473 common pathogenic *Neisseria *probes are hybridized by *N. lactamica*), only a portion of which are likely to be suitable immunological targets.

## Conclusion

Most of the genes that are considered to be virulence genes in the pathogenic *Neisseria *spp. are actually present within strains of *N. lactamica*, which is not pathogenic. The differences between these species, which make them pathogenic or not, are therefore not as great as might be supposed on the basis of their distinct behaviors and previous lists of virulence genes. Aside from genes such as the capsule biosynthesis genes, which are also not present in pathogenic *N. gonorrhoeae*, *N. lactamica *seems to only be missing a few of the genes that have been considered to-date as virulence genes. The similarity between the gene complements of the commensals and pathogens suggests that the virulence of the pathogenic *Neisseria *spp. may not lie within the genes they possess *per se*, but rather in a 'genetic personality' which is a result of the combinations of these genes, sequence variations that alter the function of gene products, the possession of genes for which a virulence phenotype has not yet been identified, and/or in differences in the regulation of genes between the species. We have recently shown that in *N. gonorrhoeae *the ability to cause disseminated gonococcal infections is dependent upon the specific combinations of genes present, rather than being due to a 'virulence' or 'invasion' gene [34]. The same may be true in the wider context of pathogenic potential and commensal colonization when *N. meningitidis *and *N. gonorrhoeae *are compared with *N. lactamica*. Without the specific combination of pathogenic-potential genes, *N. lactamica *can possess the 'virulence genes' of the pathogens, yet remain a harmless commensal.

## Methods

### Bacterial strains

Thirteen strains of *N. lactamica *(Table 1) were investigated for positive hybridization to gene probes on the pan-*Neisseria *microarray-v2. Twelve of the strains were provided by Julia Bennett and Martin Maiden from their MLST study [35] and were chosen by them as representative strains of the population structure of this species. Additional information on these strains, or representative strains from these STs, can be obtained from the *Neisseria *MLST Home Page [41]. A thirteenth strain of *N. lactamica *was obtained previously from David Stephens. This strain has been used by us in previous studies [19,20] and was added to this study to determine if additional data could be obtained from this strain. Eight commensal *Neisseria *spp. were investigated for positive hybridization to probes for genes previously reported by Stabler *et al *[17] as producing negative hybridizations in their study using the BμG@S neisserial microarray. *Neisseria cinerea*, *Neisseria elongata*, *Neisseria flavescens*, *Neisseria macacae*, *Neisseria mucosa*, *Neisseria polysaccharea*, *Neisseria sicca*, and *Neisseria subflava *were obtained from Prof. David Stephens (Emory University), which have been used in previous studies [11,19].

### The pan-*Neisseria *microarray-v2

The expanded pan-*Neisseria *microarray-v2 [6] was designed and generated using the same strategy as described previously for the pan-*Neisseria *microarray-v1 [16]. The microarray includes probes to the genome sequences of *N. gonorrhoeae *strain FA1090 (AE004969), *N. meningitidis *strains MC58 [10], Z2491 [36], and FAM18 (The Wellcome Trust Sanger Institute – unpublished), plus the *N. gonorrhoeae *strain MS11 Gonococcal Genetic Island [37], neisserial genes from GenBank/EMBL that are not in any of the genome sequences currently available (see Additional file 2 for GenBank Accessions), genes identified through our on-going investigations of MMEs [16,19] (see Additional file 2 for GenBank Accessions), and antibiotic resistance cassette markers commonly used in mutagenesis. Each probe of 150–450 bp targets the most conserved (≥ 90% identical over at least 150 bp) non-repetitive region within each coding sequence, maximizing hybridization with common probes to orthologous genes in different strains and species. Any significant cross-hybridization was eliminated, where possible, or noted, thereby minimizing the potential for cross-hybridization with other genes, which would generate false positive results. At the time this microarray was designed and constructed there was no publicly available annotation for the *N. gonorrhoeae *strain FA1090 genome sequence, therefore the microarray probes were designed using our own annotation of this sequence. This is available to view against the genome sequence, with comparisons to the other annotations, showing the probe sequences, locations, and those hybridized by *N. lactamica *at . For a more detailed description of the probe design and availability of the pan-*Neisseria *microarray, see Additional file 3. The PCR product probes generated were suspended in Genetix Spotting Solution for Amine slides and printed with non-adjacent replicates in triplicate onto Genetix Amine microarray slides using a Genetix QArray Mini microarray printer with 150 μm aQu solid pins.

### Comparative genome hybridization

Chromosomal DNA was extracted using a phenol-based method [38] from the strains listed in Table 1, following growth on GC media (BD) with Kellogg and ferric nitrate supplements [39] at 37°C, 5% CO_2 _(v/v) overnight.

20 μg of DNA was fluorescently labeled through direct incorporation of FluoroLink™ Cy3-dCTP or FluoroLink™ Cy5-dCTP (Amersham Pharmacia Biotech) using 5 units of DNA polymerase I, Klenow fragment (Bioline) and 3 μg random hexamer primers (Invitrogen) in a 50 μl reaction for 1.5 to 4 hours at 37°C. Unincorporated nucleotides and random primers were removed using QIAquick Nucleotide Removal columns (QIAGEN) according to the manufacturer's instructions.

Hybridizations were conducted in 4× SSC, 0.3% SDS under LifterSlips (Erie Scientific) at 65°C overnight. The slides were washed in 65°C 1× SSC, 0.05% SDS for 2 minutes, 0.06× SSC for 2 minutes, and fresh 0.06× SSC for 2 minutes before drying using an airbrush. Microarray slides were scanned using a ScanArray Express HT (Perkin Elmer).

Thirteen *N. lactamica *strains were assessed, twelve of which were selected for their diversity based on previous MLST analysis [40]. The thirteenth strain was provided from the collection of Prof. David Stephens (Emory University). Each strain of *N. lactamica *was hybridized to two slides, was paired with different strains in each hybridization, and was labeled with Cy3 on one slide and Cy5 on the other. This generated triads of strains and slides; for example strain 'A'-Cy3 was hybridized with strain 'B'-Cy5, strain 'B'-Cy3 with strain 'C'-Cy5, and strain 'C'-Cy3 with strain 'A'-Cy5.

### Microarray data analysis using BlueFuse

Microarray spot features were identified, quantified, and assessed using BlueFuse for Microarrays v2 (BlueGnome). Initially, all of the probes for a set of 127 previously described virulence genes for the pathogenic *Neisseria *(see Additional file 1) were visually checked for hybridization for each strain on both slides.

The complete dataset was then addressed using the BlueFuse for Microarrays (BlueGnome) pON score. The pON score non-comparatively evaluates the data from each microarray spot and uses this to report a probability of there being a hybridization signal for each spot. Unlike ratio-metric methods, this score is not dependent upon or influenced by the signal in the other channel. A pON score of zero indicates that there is no evidence for spot hybridization, while a score of one indicates that there is strong evidence that the spot has hybridized. The pON score is quantitatively calculated from two features of the data: the intensity of signal above background, and the circularity and uniformity that is characteristic of a microarray spot. First, the spot is evaluated to determine the proportion of pixels within it that are statistically inconsistent with the Bayesian calculated background noise, thus the amount of the spot that is determined to be hybridization signal. Next, since a simple signal score can be influenced by inconsistent background and other features that are not hybridized microarray spots, the characteristics of the spot feature are taken into account, i.e. whether it is round and the signal across it is uniform. This serves to discriminate between signals that are simply large, high intensity noise as opposed to those which are consistent with a hybridized microarray spot. The exact details of the calculation of this score are proprietary to BlueGnome. In CGH applications, it therefore provides an independent assessment of gene presence or absence in each channel. Each probe was printed onto the pan-*Neisseria *microarray slide in three non-adjacent locations, therefore three pON scores were available for each probe. The images were manually flagged within BlueFuse to remove all spots that were generating clearly artefactual data (such as due to dust and local hybridization effects), and the data removed from subsequent analysis. Regions of the slide were manually assessed to determine the range of pON scores that could consistently be relied upon to reflect a strong positive hybridization. Additionally, the pON scores were determined for control hybridizations to the pan-*Neisseria *microarray-v2 with the strains against which the probes were designed. For this study, the pON score for a positive probe hybridization was purposely selected to be highly stringent, at pON > 0.67, so that the genes called as present within *N. lactamica *could be relied upon to be present in this species; a lower pON score might reflect hybridization of probes to divergent alleles and orthologous genes of different function. The control hybridizations suggest that with the pathogenic *Neisseria *spp. pON scores > 0.49 are reliable, therefore the high threshold for this study should be very robust. When all three probe spot replicates had pON scores > 0.67 then the presence of that gene within the *N. lactamica *genome was accepted without further review of the slide image. For any other probes with pON scores of > 0.67 for one or two spots, the slide image was reviewed to ascertain whether hybridization was present, indicating gene presence in the strain. The results were then combined for all 13 *N. lactamica *strains. The data reported here is therefore from a pool of data that minimizes strain-specific influences on the data, either due to the absence of certain sequences in certain strains or the presence of differences within divergent gene families between strains. In all, with 8,535 probe spots on each microarray (2845 probes printed in triplicate), 17,070 probe spots were assessed per strain, for a total of 221,910 features that were evaluated in the whole study. This does not include the data for the other commensal *Neisseria *spp. hybridizations. The images for these were manually assessed for the presence of positive hybridization in the 32 gene probes that produced negative hybridizations in both our analysis of *N. lactamica *and that of Stabler *et al *[17].

## Authors' contributions

LS conducted the microarray hybridizations, analysed the microarray data, wrote the manuscript, assisted in probe design for the pan-*Neisseria *microarray, and produced the pan-*Neisseria *microarray-v2 PCR product probes. NS conceived of the pan-*Neisseria *microarray and its use in this kind of study, was the primary designer of the probes for the microarray, manufactured the pan-*Neisseria *microarray-v2 slides used in this study, supervised this work, and provided support in the preparation of the manuscript. All authors read and approved the final manuscript.

## Supplementary Material

Additional file 2 Genes from the pathogenic *Neisseria *spp. present in one or more of 13 *N. lactamica *strains. This file contains a table of all of those genes for which the associated pan-*Neisseria *microarray-v2 probe was hybridized by at least one of the *N. lactamica *strains tested. The number of strains with positive hybridizations is indicated. Click here for file

Additional file 3 Probe content and design for the pan-*Neisseria *microarray, versions 1 & 2. This file provides a detailed description of the design and construction of the pan-*Neisseria *microarray and the revised and updated pan-*Neisseria *microarray-v2. Included are details of the probe design and selection criteria, the sequences against which the microarray was designed, additions to the microarray in version 2, and publications that have used the pan-*Neisseria *microarray in CGH. Click here for file

Additional file 1 Virulence-associated and predicted virulence-associated genes from the pathogenic *Neisseri*a spp. This file contains a table of those genes from the current published literature that have been suggested to have a role in virulence. Genes for which there were positive hybridizations on the pan-*Neisseria *microarray-v2 in the *N. lactamica *strains tested are indicated, as are those genes present in all four of the currently available *Neisseria *spp. genome sequences. Click here for file
