# Supplementary material for: The majority of genes in the pathogenic Neisseria species are present in non-pathogenic Neisseria lactamica, including those designated as 'virulence genes'
Source: BMC Genomics. 2006 May 30;7:128. doi: 10.1186/1471-2164-7-128 (PMC1538595; doi:10.1186/1471-2164-7-128)
Supplement: Additional file 2 — Genes from the pathogenic Neisseria spp. present in one or more of 13 N. lactamica strains. This file contains a table of all of those genes for which the associated pan-Neisseria microarray-v2 probe was hybridized by at least one of the N. lactamica strains tested. The number of strains with positive hybridizations is indicated. [file 1471-2164-7-128-S2.pdf]

Supplementary Table 2.
Genes from the pathogenic *Neisseria* spp. present in one or more of 13 *N. lactamica* strains.

Gene[a]	Present[b]	Annotation[c]
NMB0002	9	hypothetical protein
NMB0003	10	glutamyl-tRNA synthetase (gltX)
NMB0004	6	EpiH/GdmH-related protein
NMB0005	12	arsenate reductase (arsC)
NMB0006	9	thioredoxin-related protein
NMB0007	13	cell division ATP-binding protein FtsE (ftsE)
NMB0008	10	cell division protein FtsX, putative
NMB0009	10	BolA-YrbA family protein
NMB0010	9	phosphoglycerate kinase (pgk)
NMB0011	13	UDP-N-acetylglucosamine 1-carboxyvinyltransferase (murA)
NMB0012	13	conserved hypothetical protein
NMB0014	4	3-deoxy-D-manno-octulosonic-acid transferase (kdtA)
NMB0015	6	6-phosphogluconate dehydrogenase, decarboxylating (gnd)
NMB0017	13	UDP-3-O-3-hydroxymyristoyl N-acetylglucosamine deacetylase (envA)
NMB0018	12	Type IV pilin class I (pilE)
NMB0027	7	FKBP-type peptidyl-prolyl cis-trans isomerase
NMB0028	13	hypothetical protein
NMB0029	13	glycerate dehydrogenase (hprA)
NMB0030	13	methionyl-tRNA synthetase (metG)
NMB0031	13	glucosamine--fructose-6-phosphate aminotransferase (isomerizing) (glmS)
NMB0032	13	hypothetical protein
NMB0033	12	membrane-bound lytic murein transglycosylase A, putative
NMB0034	4	conserved hypothetical protein
NMB0035	13	conserved hypothetical protein
NMB0036	10	conserved hypothetical protein
NMB0037	13	phnA protein (phnA)
NMB0038	4	UDP-N-acetylglucosamine pyrophosphorylase (glmU)
NMB0039	13	hypothetical protein
NMB0041	13	ABC transporter, periplasmic solute-binding protein
NMB0042	10	conserved hypothetical protein
NMB0043	13	conserved hypothetical protein
NMB0044	13	peptide methionine sulfoxide reductase (pilB)
NMB0045	13	signal recognition particle protein (pilA)
NMB0047	13	conserved hypothetical protein
NMB0048	1	conserved hypothetical protein
NMB0050	12	conserved hypothetical protein
NMB0051	7	twitching motility protein
NMB0052	10	twitching motility protein PilT (pilT-1)
NMB0053	5	conserved hypothetical protein
NMB0054	9	hypothetical protein
NMB0055	13	pyrroline-5-carboxylate reductase (proC)
NMB0056	13	DnaK suppressor protein (dksA)
NMB0057	13	putative DNA transport competence protein
NMB0058	13	hypothetical protein
NMB0059	10	dnaJ protein (dnaJ)
NMB0060	10	conserved hypothetical protein
NMB0061	9	dTDP-4-keto-6-deoxy-D-glucose-3,6-epimerase
NMB0062	13	glucose-1-phosphate thymidylyltransferase (rfbA-1)
NMB0063	13	dTDP-D-glucose 4,6-dehydratase (rfbB)
NMB0064	9	UDP-glucose 4-epimerase (galE)
NMB0075	9	transcriptional accessory protein Tex, putative
NMB0078	10	UDP-glucose 4-epimerase (galE)
NMB0079	13	dTDP-D-glucose 4,6-dehydratase (rfbB)
NMB0080	13	glucose-1-phosphate thymidylyltransferase (rfbA-1)
NMB0081	10	dTDP-4-keto-6-deoxy-D-glucose-3,6-epimerase
NMB0082	4	capsule polysaccharide modification protein LipA
NMB0085	10	sodium-glutamate symporter (gltS)
NMB0086	13	hypothetical protein

NMB0087	13	hypothetical protein
NMB0088	7	outer membrane protein P1, putative
NMB0089	7	pyruvate kinase II (pykA)
NMB0090	13	IS1016 family transposase
NMB0098	1	ABC transporter, ATP-binding protein
NMB0101	13	IS1016 family transposase
NMB0102	12	hypothetical protein
NMB0103	8	hypothetical protein
NMB0104	5	hypothetical protein
NMB0105	6	PhnO-related protein
NMB0106	13	aspartate carbamoyltransferase, catalytic subunit (pyrB)
NMB0107	13	aspartate carbamoyltransferase, regulatory subunit (pyrI)
NMB0108	9	hypothetical protein
NMB0109	13	conserved hypothetical protein
NMB0110	13	polypeptide deformylase (def)
NMB0111	12	methionyl-tRNA formyltransferase (fmt)
NMB0112	13	16S RNA methyltransferase (rsmB)
NMB0113	9	hypothetical protein
NMB0114	10	nitrogen regulation protein NtrY, putative
NMB0115	9	nitrogen assimilation regulatory protein NtrX (ntrX)
NMB0116	7	DNA processing chain A (dprA) [smf]
NMB0117	10	smg protein, putative
NMB0118	11	DNA topoisomerase I (topA)
NMB0121	10	conserved hypothetical protein
NMB0122	11	conserved hypothetical protein/ possible DNA methylase
NMB0123	13	ferredoxin, 4Fe-4S bacterial type
NMB0124	10	translation elongation factor Tu (tufA)
NMB0125	10	preprotein translocase subunit SecE (secE)
NMB0126	11	transcription antitermination protein NusG (nusG)
NMB0127	9	50S ribosomal protein L11 (rplK)
NMB0128	13	50S ribosomal protein L1 (rplA)
NMB0129	13	hypothetical protein
NMB0130	13	50S ribosomal protein L10 (rplJ)
NMB0131	12	50S ribosomal protein L7-L12 (rplL)
NMB0132	5	DNA-directed RNA polymerase, beta subunit (rpoB)
NMB0133	7	DNA-directed RNA polymerase, beta prime subunit (rpoC)
NMB0134	7	hypothetical protein
NMB0135	13	conserved hypothetical protein
NMB0136	13	30S ribosomal protein S12 (rpsL)
NMB0137	7	30S ribosomal protein S7 (rpsG)
NMB0138	13	elongation factor G (EF-G) (fusA)
NMB0139	10	translation elongation factor Tu (tufA)
NMB0140	13	30S ribosomal protein S10 (rpsJ)
NMB0141	9	IS1106 related transposase
NMB0142	13	50S ribosomal protein L3 (rplC)
NMB0143	13	50S ribosomal protein L4 (rplD)
NMB0144	13	50S ribosomal protein L23 (rplW)
NMB0144	12	50S ribosomal protein L23 (rplW)
NMB0145	6	50S ribosomal protein L2 (rplB)
NMB0146	10	30S ribosomal protein S19 (rpsS)
NMB0147	13	50S ribosomal protein L22 (rplV)
NMB0148	13	30S ribosomal protein S3 (rpsC)
NMB0149	13	50S ribosomal protein L16 (rplP)
NMB0150	13	50S ribosomal protein L29 (rpmC)
NMB0151	9	30S ribosomal protein S17 (rpsQ)
NMB0152	12	50S ribosomal protein L14 (rplN)
NMB0153	13	50S ribosomal protein L24 (rplX)
NMB0154	12	50S ribosomal protein L5 (rplE)
NMB0155	13	30S ribosomal protein S14 (rpsN)

NMB0156	13	30S ribosomal protein S8 (rpsH)
NMB0157	13	50S ribosomal protein L6 (rplF)
NMB0158	13	50S ribosomal protein L18 (rplR)
NMB0159	13	30s ribosomal protein S5 (rpsE)
NMB0160	13	50S ribosomal protein L30 (rpl30)
NMB0161	8	50S ribosomal protein L15 (rplO)
NMB0162	11	preprotein translocase SecY subunit (secY)
NMB0163	10	translation initiation factor IF-1 (infA)
NMB0164	4	50S ribosomal protein L36 (rpmJ)
NMB0165	13	30S ribosomal protein S13 (rpsM)
NMB0167	13	30S ribosomal protein S4 (rpsD)
NMB0168	13	DNA-directed RNA polymerase, alpha subunit (rpoA)
NMB0169	13	50S ribosomal protein L17 (rplQ)
NMB0170	13	septum site-determining protein MinC (minC)
NMB0171	13	septum site-determining protein MinD (minD)
NMB0172	5	cell division topological specificity factor (minE)
NMB0173	13	transcriptional regulator, LysR family
NMB0174	12	valyl-tRNA synthetase (valS)
NMB0178	10	acyl-(acyl-carrier-protein)--UDP-N-acetylglucosamine O-acyltransferase (lpxA)
NMB0179	13	(3R)-hydroxymyristoyl-(acyl carrier protein) dehydratase (fabZ)
NMB0180	12	UDP-3-O-(3-hydroxymyristoyl)-glucosamine N-acyltransferase (lpxD)
NMB0181	13	outer membrane protein OmpH, putative
NMB0182	11	outer membrane protein Omp85 (omp85)
NMB0183	2	conserved hypothetical protein
NMB0184	13	1-deoxy-D-xylulose 5-phosphate reductoisomerase (dxr)
NMB0185	12	phosphatidate cytidylyltransferase (cdsA)
NMB0186	13	undecaprenyl pyrophosphate synthetase (uppS)
NMB0187	12	ribosome recycling factor (frr)
NMB0188	8	conserved hypothetical protein
NMB0189	9	hypothetical protein
NMB0190	11	glucose inhibited division protein B (gidB)
NMB0191	10	ParA family protein
NMB0192	11	ribonuclease HII (rnhB)
NMB0193	13	glucose inhibited division protein A (gidA)
NMB0194	10	amino acid symporter, putative
NMB0195	6	pyridoxal phosphate biosynthetic protein PdxA (pdxA)
NMB0196	13	ribonuclease E (rne)
NMB0197	6	hypothetical protein
NMB0198	13	ribosomal large subunit pseudouridine synthase C (rluC)
NMB0199	13	lipid-A-disaccharide synthase (lpxB)
NMB0200	13	hypothetical protein
NMB0201	13	hypothetical protein
NMB0202	13	hypothetical protein
NMB0203	5	dihydrodipicolinate reductase (dapB)
NMB0204	6	lipoprotein, putative (smpA)
NMB0205	11	ferric uptake regulation protein (fur)
NMB0206	10	leucyl-phenylalanyl-tRNA--protein transferase (aat)
NMB0207	13	glyceraldehyde 3-phosphate dehydrogenase (gapA-1)
NMB0208	13	ferredoxin, 4Fe-4S bacterial type
NMB0209	12	glutathione-regulated potassium-efflux system protein (kefB)
NMB0210	13	modification methylase
NMB0211	12	L-serine dehydratase (sdaA)
NMB0212	6	DNA gyrase subunit B (gyrB)
NMB0213	10	hypothetical protein
NMB0214	3	oligopeptidase A (prlC)
NMB0215	12	conserved hypothetical protein
NMB0216	1	catalase (kat)
NMB0217	1	RNA polymerase sigma-54 factor RpoN, putative
NMB0218	6	glycosyltransferase (pglA)

NMB0219	4	3-oxoacyl-(acyl-carrier-protein) synthase II (fabF-1)
NMB0220	12	acyl carrier protein (acp-1)
NMB0221	4	dihydroorotate dehydrogenase (pyrD)
NMB0222	13	hypothetical protein
NMB0223	11	hypothetical protein
NMB0224	9	glutamate-ammonia-ligase adenylyltransferase (glnE)
NMB0232	7	DNA helicase II (uvrD)
NMB0234	13	hypothetical protein
NMB0235	13	hypothetical protein
NMB0237	13	hypothetical protein
NMB0238	13	IS1016 family transposase
NMB0239	13	hypothetical protein
NMB0240	6	hypothetical protein
NMB0241	13	NADH dehydrogenase I, A subunit (nuoA)
NMB0242	9	NADH dehydrogenase I, B subunit (nuoB)
NMB0243	6	NADH dehydrogenase I, C subunit (nuoC)
NMB0244	6	NADH dehydrogenase I, D subunit (nuoD)
NMB0245	11	NADH dehydrogenase I, E subunit (nuoE)
NMB0246	11	NADH dehydrogenase I, F subunit (nuoF)
NMB0247	7	hypothetical protein
NMB0249	8	NADH dehydrogenase I, G subunit (nuoG)
NMB0250	13	NADH dehydrogenase I, H subunit (nuoH)
NMB0251	13	NADH dehydrogenase I, I subunit (nuoI)
NMB0253	7	NADH dehydrogenase I, J subunit (nuoJ)
NMB0254	10	NADH dehydrogenase I, K subunit (nuoK)
NMB0255	5	cell filamentation protein Fic-related protein
NMB0256	6	hypothetical protein
NMB0257	8	NADH dehydrogenase I, L subunit (nuoL)
NMB0258	6	NADH dehydrogenase I, M subunit (nuoM)
NMB0259	12	NADH dehydrogenase I, N subunit (nuoN)
NMB0260	10	hypothetical protein
NMB0261	9	geranyltranstransferase (ispA)
NMB0262	8	exodeoxyribonuclease, small subunit (xseB)
NMB0263	7	conserved hypothetical protein
NMB0264	6	ABC transporter, ATP-binding protein
NMB0265	13	Holliday junction DNA helicase RuvA (ruvA)
NMB0266	10	conserved hypothetical protein
NMB0267	13	conserved hypothetical protein
NMB0268	9	RNA methyltransferase, TrmH family
NMB0269	3	bioH protein, putative
NMB0270	9	bioH protein, putative
NMB0271	10	hypothetical protein
NMB0272	11	hypothetical protein
NMB0274	13	ATP-dependent DNA helicase RecQ (recQ)
NMB0275	13	indole-3-glycerol phosphate synthase (trpC)
NMB0276	13	conserved hypothetical protein
NMB0277	13	virulence factor MviN (mviN)
NMB0278	13	thiol:disulfide interchange protein DsbA (dsbA-1)
NMB0279	12	conserved hypothetical protein
NMB0280	7	organic solvent tolerance protein, putative
NMB0281	10	peptidyl-prolyl cis-trans isomerase
NMB0282	13	ribonuclease II-related protein
NMB0283	13	conserved hypothetical protein
NMB0284	13	adenylosuccinate lyase (purB)
NMB0284	13	adenylosuccinate lyase (purB)
NMB0286	10	conserved hypothetical protein
NMB0287	13	probable ATP-dependent helicase DinG (dinG)
NMB0288	13	hypothetical protein
NMB0289	8	deoxyribodipyrimidine photolyase, (phrB)

NMB0290	12	transcriptional regulator, putative
NMB0291	12	conserved hypothetical protein
NMB0292	13	conserved hypothetical protein
NMB0294	9	thiol:disulfide interchange protein DsbA
NMB0295	11	signal recognition particle protein (ffh)
NMB0296	13	CcsA-related protein
NMB0296	13	CcsA-related protein
NMB0297	7	hypothetical protein
NMB0299	13	putative DNA transport competence protein
NMB0302	13	IS1016 transposase
NMB0305	1	hypothetical protein
NMB0307	13	phospho-2-dehydro-3-deoxyheptonate aldolase, phe-sensitive (aroG)
NMB0308	7	dihydrofolate reductase (folA)
NMB0309	12	conserved hypothetical protein
NMB0310	4	conserved hypothetical protein
NMB0311	6	hypothetical protein
NMB0312	6	vapA
NMB0314	9	hypothetical protein
NMB0315	6	conserved hypothetical protein
NMB0316	7	conserved hypothetical protein
NMB0317	10	conserved hypothetical protein
NMB0318	9	fatty acid efflux system protein (farA)
NMB0319	9	fatty acid efflux system protein (farB)
NMB0320	11	hypothetical protein
NMB0321	10	50S ribosomal protein L28 (rpmB)
NMB0322	13	50S ribosomal protein L33 (rpmG)
NMB0323	13	UbiH family protein
NMB0324	13	50S ribosomal protein L27 (rpmA)
NMB0325	13	50S ribosomal protein L21 (rplU)
NMB0326	9	octaprenyl-diphosphate synthase (ispB)
NMB0327	9	conserved hypothetical protein
NMB0329	13	type IV pilus assembly protein (pilF)
NMB0330	10	conserved hypothetical protein
NMB0333	13	pilus assembly protein PilG (pilG)
NMB0334	12	glucose-6-phosphate isomerase (pgi-1)
NMB0335	2	2,3,4,5-tetrahydropyridine-2-carboxylate N-succinyltransferase (dapD)
NMB0336	12	enoyl-(acyl-carrier-protein) reductase (fabI)
NMB0337	6	branched-chain amino acid aminotransferase, putative
NMB0338	12	hypothetical protein
NMB0339	7	conserved hypothetical protein
NMB0341	13	TspA protein
NMB0342	13	intracellular septation protein A (ispA)
NMB0343	9	conserved hypothetical protein
NMB0344	12	BolA-YrbA family protein
NMB0345	13	cell-binding factor, putative
NMB0346	13	hypothetical protein
NMB0347	13	conserved hypothetical protein
NMB0348	13	conserved hypothetical protein
NMB0349	12	glutamyl-tRNA synthetase-related protein
NMB0350	12	hypothetical protein
NMB0351	10	transaldolase (tal)
NMB0352	13	sugar isomerase, KpsF-GutQ family
NMB0353	13	conserved hypothetical protein
NMB0354	13	hypothetical protein
NMB0355	12	conserved hypothetical protein
NMB0356	5	ABC transporter, ATP-binding protein
NMB0357	12	monofunctional biosynthetic peptidoglycan transglycosylase (mtgA)
NMB0358	13	shikimate 5-dehydrogenase (aroE)
NMB0359	13	glutamate--ammonia ligase (glnA)

NMB0360	13	AmpG-related protein
NMB0361	13	conserved hypothetical protein
NMB0362	13	hypothetical protein
NMB0364	10	FrpC operon protein
NMB0365	10	iron-regulated protein FrpC
NMB0366	13	hypothetical protein
NMB0367	13	hypothetical protein
NMB0368	12	hypothetical protein
NMB0370	13	hypothetical protein
NMB0371	13	hypothetical protein
NMB0373	7	hypothetical protein
NMB0374	13	MafB-related protein
NMB0375	10	mafA protein (mafA-2)
NMB0376	9	hypothetical protein
NMB0377	7	conserved hypothetical protein
NMB0378	10	phosphate permease, putative
NMB0379	11	oxygen-independent coproporphyrinogen III oxidase (hemN)
NMB0381	13	cys regulon transcriptional activator (cysB)
NMB0382	13	outer membrane protein class 4 (rmpM)
NMB0383	12	hypothetical protein
NMB0384	13	hypothetical protein
NMB0385	7	thiamin-monophosphate kinase (thiL)
NMB0386	7	phosphatidylglycerophosphatase A (pgpA)
NMB0387	9	ABC transporter, ATP-binding protein
NMB0388	13	sugar transporter, putative
NMB0388	10	sugar transporter, putative
NMB0389	10	aldose 1-epimerase (galM)
NMB0390	6	maltose phosphorylase (mapA)
NMB0391	7	beta-phosphoglucomutase (pgmB)
NMB0392	4	l-aspartate oxidase (nadB)
NMB0394	7	quinolinate synthetase A (nadA)
NMB0395	12	conserved hypothetical protein
NMB0396	13	nicotinate-nucleotide pyrophosphorylase (nadC)
NMB0397	7	hypothetical protein
NMB0398	13	transcriptional regulator, ArsR family
NMB0399	13	exodeoxyribonuclease III (xthA)
NMB0400	8	transposase
NMB0401	11	proline dehydrogenase (putA)
NMB0402	13	sodium/proline symporter (putP)
NMB0403	9	hypothetical protein
NMB0404	13	conserved hypothetical protein
NMB0405	13	competence protein ComM (comM)
NMB0407	7	thiol:disulfide interchange protein DsbA (dsbA-3)
NMB0408	12	bacitracin resistance protein (bacA)
NMB0409	13	conserved hypothetical protein
NMB0410	13	conserved hypothetical protein
NMB0411	7	conserved hypothetical protein
NMB0412	9	cell division protein FtsL-related protein
NMB0413	13	penicillin-binding protein 2 (penA)
NMB0414	13	UDP-N-acetylmuramoylalanyl-D-glutamate--2,6-diaminopimelate ligase (murE)
NMB0416	7	UDP-N-acetylmuramoylalanyl-D-glutamyl-2,6-diaminopimelate--D-alanyl-D-alanyl ligase (murF)
NMB0417	13	hypothetical protein (dcaB)
NMB0418	13	phospho-N-acetylmuramoyl-pentapeptide-transferase (mraY)
NMB0419	11	conserved hypothetical protein (dcaC)
NMB0420	13	UDP-N-acetylmuramoylalanine--D-glutamate ligase (murD)
NMB0421	13	cell division protein FtsW (ftsW)
NMB0422	12	cell division transferase protein MurG (murG)
NMB0423	12	UDP-N-acetylmuramate--alanine ligase (murC)
NMB0424	12	D-alanine--D-alanine ligase (ddlB)

NMB0425	7	cell division protein FtsQ (ftsQ)
NMB0426	3	cell division protein FtsA (ftsA)
NMB0427	13	cell division protein FtsZ (ftsZ)
NMB0428	13	conserved hypothetical protein
NMB0436	13	conserved hypothetical protein
NMB0437	13	conserved hypothetical protein
NMB0438	10	hypothetical protein
NMB0439	13	conserved hypothetical protein
NMB0440	11	prephenate dehydrogenase, putative
NMB0442	13	opacity protein (opa)
NMB0444	12	conserved hypothetical protein
NMB0445	13	bicyclomycin resistance protein, putative
NMB0447	8	DNA repair protein RecO (recO)
NMB0448	13	pyridoxal phosphate biosynthetic protein PdxJ (pdxJ)
NMB0452	13	holo-(acyl-carrier protein) synthase (acpS)
NMB0453	13	mutT protein (mutT)
NMB0454	13	hypothetical protein
NMB0455	13	conserved hypothetical protein
NMB0456	12	N-acetylmuramoyl-L-alanine amidase (amiC)
NMB0457	9	conserved hypothetical protein
NMB0458	11	glutamate racemase (murI)
NMB0459	13	conserved hypothetical protein
NMB0460	13	transferrin-binding protein 2 (tbp2)
NMB0461	12	transferrin-binding protein 1 (tbp1)
NMB0462	13	spermidine/putrescine ABC transporter, periplasmic spermidine/putrescine-binding protein (potD-1)
NMB0463	12	30S ribosomal protein S20 (rpsT)
NMB0464	7	phospholipase A1, putative
NMB0465	13	conserved hypothetical protein
NMB0466	9	aspartyl-tRNA synthetase (aspS)
NMB0467	13	hypothetical protein
NMB0475	13	hypothetical protein
NMB0476	10	hypothetical protein
NMB0477	13	conserved hypothetical protein
NMB0478	13	hypothetical protein
NMB0479	13	conserved hypothetical protein
NMB0483	6	putative integral membrane protein
NMB0488	10	hypothetical protein
NMB0490	12	PspA-related protein
NMB0491	12	hypothetical protein
NMB0492	12	hypothetical protein
NMB0493	13	hemagglutinin/hemolysin-related protein
NMB0494	12	DNA helicase, truncation
NMB0495	11	replication protein
NMB0512	13	hypothetical protein
NMB0524	7	ribonuclease BN, putative
NMB0525	7	aluminum resistance protein, putative
NMB0526	13	hypothetical protein
NMB0527	13	6-pyruvoyl tetrahydrobiopterin synthase, putative
NMB0528	13	conserved hypothetical protein
NMB0529	13	conserved hypothetical protein
NMB0530	8	glycosyl hydrolase, family 3
NMB0531	12	conserved hypothetical protein
NMB0532	7	DO like serine protease (htrA family)
NMB0533	12	endonuclease III (nth)
NMB0534	13	conserved hypothetical protein
NMB0536	6	Na\+/H\+ antiporter
NMB0537	13	conserved hypothetical protein
NMB0538	13	conserved hypothetical protein
NMB0539	13	porphobilinogen deaminase (hemC)

NMB0540	7	aspartate aminotransferase (aspC)
NMB0542	9	hypothetical protein
NMB0543	4	L-lactate permease, putative
NMB0544	7	conserved hypothetical protein
NMB0545	8	conserved hypothetical protein
NMB0546	8	alcohol dehydrogenase, propanol-preferring (adhP)
NMB0547	13	type IV pilin protein (pilV)
NMB0548	12	AcrA-AcrE family protein
NMB0549	13	ABC transporter, ATP-binding protein (ybjZ)
NMB0550	13	thiol:disulfide interchange protein DsbC
NMB0551	12	primosomal protein n` (priA)
NMB0552	10	hypothetical protein
NMB0553	8	transposase
NMB0554	13	dnaK protein (dnaK)
NMB0555	13	hypothetical protein
NMB0556	12	repressor protein, putative
NMB0557	13	conserved hypothetical protein
NMB0558	12	hypothetical protein
NMB0559	13	ubiquinone biosynthesis protein AarF (aarF)
NMB0560	13	serine acetyltransferase (cysE)
NMB0561	13	grpE protein (grpE)
NMB0562	13	conserved hypothetical protein
NMB0563	13	thiamine biosynthesis lipoprotein ApbE (apbE)
NMB0564	13	Na(\+)-translocating NADH-quinone reductase, subunit F (nqrF)
NMB0565	13	Na(\+)-translocating NADH-quinone reductase, subunit E (nqrE)
NMB0566	12	Na(\+)-translocating NADH-quinone reductase, subunit D (nqrD)
NMB0567	13	Na(\+)-translocating NADH-quinone reductase, subunit C (nqrC)
NMB0568	12	Na(\+)-translocating NADH-quinone reductase, subunit B (nqrB)
NMB0569	12	Na(\+)-translocating NADH-quinone reductase, subunit A (nqrA)
NMB0570	12	hypothetical protein
NMB0571	13	conserved hypothetical protein
NMB0572	9	hypothetical protein
NMB0573	13	transcriptional regulator, AsnC family
NMB0574	13	glycine cleavage system T protein (gcvT)
NMB0575	13	glycine cleavage system H protein (gcvH)
NMB0576	13	glutamyl-tRNA reductase (hemA)
NMB0577	13	regulatory protein (nosR)
NMB0578	13	copper ABC transporter, periplasmic copper-binding protein (nosD)
NMB0579	10	copper ABC transporter, ATP-binding protein (nosF)
NMB0580	13	protein disulfide isomerase NosL, putative
NMB0581	13	electron transfer flavoprotein-ubiquinone oxidoreductase
NMB0582	12	bacteriocin resistance protein, putative
NMB0583	13	IS1016 transposase
NMB0584	10	FrpC operon protein
NMB0586	12	periplasmic binding protein for ABC transporter
NMB0587	7	membrane protein
NMB0588	9	ABC transporter, ATP-binding protein
NMB0589	13	50s ribosomal protein L19 (rplS)
NMB0590	10	tRNA (guanine-N1)-methyltransferase (trmD)
NMB0591	7	16S rRNA processing protein RimM (rimM)
NMB0592	7	30S ribosomal protein S16 (rpsP)
NMB0593	7	conserved hypothetical protein (dead)
NMB0594	9	sensor histidine kinase
NMB0595	3	DNA-binding response regulator
NMB0596	4	hypothetical protein
NMB0597	4	hypothetical protein
NMB0598	12	Maf-YceF-YhdE family protein
NMB0599	8	conserved hypothetical protein
NMB0600	10	hypothetical protein

NMB0601	11	conserved hypothetical protein
NMB0602	9	hitA protein (hitA)
NMB0603	2	phosphoribosyl-ATP cyclohydrolase (hisE)
NMB0604	12	alcohol dehydrogenase, zinc-containing
NMB0605	6	histone deacetylase family protein
NMB0607	6	protein-export membrane protein SecD (secD)
NMB0608	10	protein-export membrane protein SecF (secF)
NMB0609	9	30s ribosomal protein S15 (rpsO)
NMB0610	12	spermidine/putrescine ABC transporter, ATP-binding protein (potA-1)
NMB0611	11	spermidine/putrescine ABC transporter, permease protein (potB)
NMB0612	7	spermidine/putrescine ABC transporter, permease protein (potC)
NMB0613	9	hypothetical protein
NMB0614	10	oxidoreductase, putative
NMB0615	11	ammonium transporter AmtB, putative
NMB0616	13	IS1016 family transposase
NMB0617	6	transcription termination factor Rho (rho)
NMB0618	8	phosphoenolpyruvate synthase (ppsA)
NMB0619	5	conserved hypothetical protein
NMB0620	8	phosphoglycolate phosphatase (pgp)
NMB0621	6	conserved hypothetical protein
NMB0622	6	outer membrane lipoprotein carrier protein (lolA)
NMB0623	13	spermidine/putrescine ABC transporter, periplasmic spermidine/putrescine-binding protein (potD-2)
NMB0624	13	glycosyltransferase
NMB0625	2	conserved hypothetical protein
NMB0626	7	peptide chain release factor 3 (prfC)
NMB0627	12	phosphoribosyl-AMP cyclohydrolase (hisI)
NMB0628	8	hisF protein (hisF)
NMB0629	8	phosphotransacetylase, putative
NMB0630	10	amidotransferase HisH (hisH)
NMB0631	10	phosphate acetyltransferase Pta, putative
NMB0632	5	iron(III) ABC transporter, ATP-binding protein (fbpC)
NMB0633	13	iron(III) ABC transporter, permease protein (fbpB)
NMB0634	13	iron(III) ABC transporter, periplasmic binding protein (fbpA)
NMB0636	13	hypothetical protein
NMB0637	9	argininosuccinate lyase (argH)
NMB0638	13	UTP--glucose-1-phosphate uridylyltransferase (galU)
NMB0639	12	conserved hypothetical protein
NMB0640	13	hypothetical protein
NMB0641	13	inorganic pyrophosphatase (ppa)
NMB0642	13	dATP pyrophosphohydrolase (ntpA)
NMB0643	12	MafB adhesion protein
NMB0644	12	hypothetical protein
NMB0646	13	ribonuclease inhibitor barstar
NMB0648	13	hypothetical protein
NMB0649	12	hypothetical protein
NMB0650	13	hypothetical protein
NMB0651	13	hypothetical protein
NMB0652	10	mafA protein (mafA-2)
NMB0653	13	MafB-related protein
NMB0656	2	hypothetical protein
NMB0657	12	MafB related sequence
NMB0658	12	MafB related sequence
NMB0659	11	hypothetical protein
NMB0660	13	hypothetical protein
NMB0661	9	transmembrane potassium transporter (trkH)
NMB0662	13	hypothetical protein
NMB0663	8	outer membrane protein NsgA (nsgA)
NMB0664	3	hypothetical protein
NMB0665	12	oxygen-independent coprophorphyrinogen III oxidase family protein

NMB0666	6	DNA ligase (ligA-1)
NMB0667	13	hypothetical protein
NMB0668	12	ampD protein (ampD)
NMB0669	4	conserved hypothetical protein
NMB0670	9	thymidylate kinase (tmk)
NMB0671	12	malate oxidoreductase (NAD) (sfcA)
NMB0672	9	tetraacyldisaccharide 4`-kinase (lpxK)
NMB0673	7	hypothetical protein
NMB0674	13	conserved hypothetical protein
NMB0675	13	3-deoxy-D-manno-octulosonate cytidylyltransferase (kdsB)
NMB0676	10	hypothetical protein
NMB0678	12	tryptophan synthase, alpha subunit (trpA)
NMB0679	12	acetyl-CoA carboxylase, carboxyl transferase beta subunit (accD)
NMB0680	7	cryptic protein (cnp1)
NMB0681	9	conserved hypothetical protein
NMB0682	7	dihydroorotase (pyrC)
NMB0683	9	N utilization substance protein B (nusB)
NMB0685	13	hypothetical protein
NMB0686	7	ribonuclease III (rnc)
NMB0687	12	GTP-binding protein Era (era)
NMB0688	12	N-(5 prime -phosphoribosyl)anthranilate isomerase (trpF)
NMB0689	13	transcription elongation factor GreB (greB)
NMB0690	13	amidophosphoribosyltransferase (purF)
NMB0691	7	colicin V production protein, putative
NMB0692	4	tpc protein (tpc)
NMB0693	3	folylpolyglutamate synthase-dihydrofolate synthase (folC)
NMB0694	6	folI protein (folI)
NMB0695	10	hypothetical protein
NMB0696	12	amino acid ABC transporter, ATP-binding protein
NMB0697	12	dimethyladenosine transferase (ksgA)
NMB0698	13	hypothetical protein
NMB0699	10	tryptophan synthase, beta subunit (trpB)
NMB0702	7	competence protein ComA (comA)
NMB0703	12	competence lipoprotein ComL (comL)
NMB0704	6	ribosomal large subunit pseudouridine synthase D (rluD)
NMB0705	8	transporter
NMB0706	9	conserved hypothetical protein
NMB0707	13	rare lipoprotein B, putative
NMB0708	12	DNA polymerase III, delta subunit (holA)
NMB0709	13	Hypothetical protein
NMB0710	12	Hypothetical protein
NMB0711	12	conserved hypothetical protein
NMB0712	13	RNA polymerase sigma-32 factor (rpoH)
NMB0713	13	apolipoprotein N-acyltransferase, putative
NMB0717	12	cytochrome, putative
NMB0718	12	ferrochelatase (hemH)
NMB0719	13	queuine tRNA-ribosyltransferase (tgt)
NMB0720	10	threonyl-tRNA synthetase (thrS)
NMB0721	12	translation initiation factor 3 (infC)
NMB0722	13	50S ribosomal protein L35 (rpmI)
NMB0723	12	50S ribosomal protein L20 (rplT)
NMB0724	7	phenylalanyl-tRNA synthetase, alpha chain (pheS)
NMB0728	13	phenylalanyl-tRNA synthetase, beta chain (pheT)
NMB0729	13	integration host factor, alpha subunit (himA)
NMB0732	13	adenosylmethionine-8-amino-7-oxononanoate aminotransferase (bioA)
NMB0733	13	dethiobiotin synthase (bioD)
NMB0734	13	hypothetical protein
NMB0735	13	4-hydroxybenzoate octaprenyltransferase (ubiA)
NMB0736	10	PTS system, nitrogen regulatory IIA protein (ptsN)

NMB0737	5	HPr kinase/phosphatase, putative
NMB0738	13	conserved hypothetical protein
NMB0739	12	conserved hypothetical protein
NMB0740	13	DNA repair protein RecN (recN)
NMB0741	13	conserved hypothetical protein
NMB0742	13	conserved hypothetical protein
NMB0743	13	ubiquinone/menaquinone biosynthesis methlytransferase UbiE (ubiE)
NMB0744	13	hypothetical protein
NMB0745	13	2-amino-4-hydroxy-6-hydroxymethyldihydropteridine-pyrophosphokinase (folK)
NMB0746	11	conserved hypothetical protein
NMB0747	5	conserved hypothetical protein
NMB0748	7	host factor-I (hfq)
NMB0749	11	penicillin-binding protein 4
NMB0750	7	bacterioferritin comigratory protein (bcp)
NMB0751	2	integrase/recombinase XerD (xerD)
NMB0752	3	bacterioferritin-associated ferredoxin, putative
NMB0753	3	conserved hypothetical protein
NMB0756	9	dTDP-L-rhamnose synthase, putative
NMB0757	11	phosphoribosylaminoimidazole-succinocarboxamide synthase (purC)
NMB0758	9	polyribonucleotide nucleotidyltransferase (pnp)
NMB0759	10	conserved hypothetical protein
NMB0760	10	diaminopimelate epimerase (dapF)
NMB0761	12	hypothetical protein
NMB0762	9	hypothetical protein
NMB0763	12	cysteine synthase (cysK)
NMB0764	9	conserved hypothetical protein
NMB0765	12	signal peptidase I (lepB)
NMB0766	13	GTP-binding protein LepA (lepA)
NMB0767	13	5-methylthioadenosine nucleosidase-S-adenosylhomocysteine nucleosidase (pfs)
NMB0768	8	twitching motility protein PilT (pilT-2)
NMB0769	6	DNA polymerase III, delta prime subunit, putative
NMB0770	8	type IV pilus assembly protein PilZ, putative
NMB0771	7	conserved hypothetical protein
NMB0773	7	conserved hypothetical protein
NMB0774	6	uracil phosphoribosyltransferase (upp)
NMB0775	6	hypothetical protein
NMB0776	9	conserved hypothetical protein
NMB0777	12	uroporphyrinogen-III synthase HemD, putative
NMB0778	7	uroporphyrin-III C-methyltransferase HemX, putative
NMB0779	7	hypothetical protein
NMB0780	12	hypothetical protein
NMB0781	7	uroporphyrinogen decarboxylase (hemE)
NMB0782	10	DNA repair protein RadA (radA)
NMB0783	10	conserved hypothetical protein
NMB0784	13	phage shock protein E precursor, putative
NMB0785	7	exodeoxyribonuclease V 135 KD polypeptide (recB)
NMB0786	8	conserved hypothetical protein
NMB0787	13	amino acid ABC transporter, periplasmic amino acid-binding protein
NMB0788	13	amino acid ABC transporter, permease protein
NMB0789	13	amino acid ABC transporter, ATP-binding protein
NMB0790	13	phosphoglucomutase (pgm)
NMB0791	12	peptidyl-prolyl cis-trans isomerase
NMB0792	9	transporter, NadC family
NMB0793	13	hypothetical protein
NMB0794	1	hypothetical protein
NMB0795	13	peptidyl-tRNA hydrolase (pth)
NMB0796	13	conserved hypothetical protein
NMB0797	13	conserved hypothetical protein
NMB0798	13	cell division protein FtsH (ftsH)

NMB0799	13	cell division protein FtsJ (ftsJ)
NMB0800	12	conserved hypothetical protein
NMB0801	13	delta-aminolevulinic acid dehydratase (hemB)
NMB0802	13	cystathionine gamma-synthase (metB)
NMB0803	13	conserved hypothetical protein
NMB0804	10	NAD(P)H nitroreductase, putative
NMB0806	10	conserved hypothetical protein
NMB0807	13	conserved hypothetical protein
NMB0808	13	hypothetical protein
NMB0809	10	conserved hypothetical protein
NMB0811	10	UDP-N-acetylpyruvoylglucosamine reductase (murB)
NMB0812	13	conserved hypothetical protein
NMB0814	12	histidyl-tRNA synthetase (hisS-1)
NMB0815	5	adenylosuccinate synthetase (purA)
NMB0822	11	heat shock protein HtpX (htpX)
NMB0823	10	adenylate kinase (adk)
NMB0824	8	orotidine 5`-phosphate decarboxylase (pyrF)
NMB0825	13	ADP-heptose synthase, putative
NMB0826	12	C-5 cytosine-specific DNA methylase
NMB0827	12	type II restriction enzyme-related protein
NMB0828	10	ADP-L-glycero-D-mannoheptose-6-epimerase (rfaD)
NMB0829	6	type I restriction enzyme EcoR124II M protein (hsdM)
NMB0832	4	anticodon nuclease
NMB0835	8	type I restriction enzyme EcoR124II R protein, putative
NMB0836	13	ATP-dependent Clp protease, ATP-binding subunit ClpA (clpA)
NMB0837	13	conserved hypothetical protein
NMB0838	7	transcriptional regulator, possibly cold shock related
NMB0839	3	pmbA protein (pmbA)
NMB0840	10	conserved hypothetical protein
NMB0841	7	hypothetical protein
NMB0842	7	single-stranded-DNA-specific exonuclease RecJ (recJ)
NMB0843	10	polyA polymerase (pcnB)
NMB0845	10	PhoH-related protein
NMB0848	9	hypothetical protein
NMB0849	13	deoxycytidine triphosphate deaminase, putative
NMB0850	13	hypothetical protein
NMB0851	7	recombination associated protein RdgC (rdgC)
NMB0852	3	essential GTPase
NMB0853	6	conserved hypothetical protein
NMB0854	10	histidyl-tRNA synthetase (hisS-2)
NMB0855	12	bacteriocin resistance protein, putative
NMB0862	5	hypothetical protein
NMB0863	6	hypothetical protein
NMB0864	6	hypothetical protein
NMB0865	10	hypothetical protein
NMB0866	13	hypothetical protein
NMB0867	9	YabO/YceC/SfhB family protein
NMB0868	5	conserved hypothetical protein
NMB0869	13	hypothetical protein
NMB0870	13	3-methyl-2-oxobutanoate hydroxymethyltransferase (panB)
NMB0871	12	pantoate--beta-alanine ligase (panC)
NMB0872	7	conserved hypothetical protein
NMB0873	9	outer membrane lipoprotein LolB, putative
NMB0874	13	conserved hypothetical protein
NMB0875	9	ribose-phosphate pyrophosphokinase (prsA)
NMB0876	13	50S ribosomal protein L25 (rplY)
NMB0877	10	penicillin-binding protein
NMB0878	12	threonine dehydratase (ilvA)
NMB0879	9	sulfate ABC transporter, ATP-binding protein (cysA)

NMB0880	11	sulfate ABC transporter, permease protein (cysW)
NMB0881	9	sulfate ABC transporter, permease protein (cysT)
NMB0882	7	hypothetical protein
NMB0884	13	superoxide dismutase (sodB)
NMB0885	10	replicative DNA helicase (dnaB)
NMB0886	11	fimbrial protein FimT / PilH (pilH)
NMB0887	13	type IV pilus assembly protein PilI (pilI)
NMB0888	13	type IV pilin-related protein PilJ (pilJ)
NMB0889	13	type IV pilin-related protein PilK (pilK)
NMB0890	13	type IV pilin-related protein PilL / PilX (pill)
NMB0892	11	AzlC-related protein
NMB0893	10	deoxyuridine 5`-triphosphate nucleotidohydrolase (dut)
NMB0894	13	aminotransferase, class I
NMB0895	11	conserved hypothetical protein
NMB0897	9	hypothetical protein
NMB0898	5	hypothetical protein
NMB0899	4	hypothetical protein
NMB0902	3	hypothetical protein
NMB0903	3	hypothetical protein
NMB0910	7	transcriptional regulator
NMB0917	6	death-on-curing protein
NMB0918	6	hypothetical protein
NMB0919	13	IS1106 transposase
NMB0920	13	isocitrate dehydrogenase (icd)
NMB0921	7	hypothetical protein
NMB0922	13	alpha-2,3-sialyltransferase
NMB0923	9	cytochrome c
NMB0924	10	oxidoreductase, short-chain dehydrogenase-reductase family
NMB0925	8	acyl CoA thioester hydrolase family protein
NMB0926	13	opacity protein (opa)
NMB0927	12	proline iminopeptidase (pip)
NMB0928	9	hypothetical protein
NMB0929	5	dihydrodipicolinate synthase (dapA)
NMB0930	9	xanthine-uracil permease family protein
NMB0931	1	RNA methyltransferase, TrmH family
NMB0933	9	cytidine and deoxycytidylate deaminase family protein
NMB0935	6	tRNA delta(2)-isopentenylpyrophosphate transferase (miaA)
NMB0936	10	hypothetical protein
NMB0937	8	elongation factor P (EF-P) (efp)
NMB0938	8	hypothetical protein
NMB0939	7	conserved hypothetical protein
NMB0940	13	homoserine O-acetyltransferase
NMB0941	13	50S ribosomal protein L36 (rpmJ)
NMB0942	13	50S ribosomal protein L31, putative (rpmE)
NMB0943	13	5,10-methylenetetrahydrofolate reductase (metF)
NMB0944	11	5-methyltetrahydropteroyltriglutamate-homocysteine methyltransferase (metH)
NMB0945	8	hypothetical protein
NMB0946	13	peroxiredoxin 2 family protein-glutaredoxin
NMB0947	10	lipoamide dehydrogenase, putative
NMB0948	6	succinate dehydrogenase, cytochrome b556 subunit (sdhC)
NMB0949	13	succinate dehydrogenase, hydrophobic membrane anchor protein (sdhD)
NMB0950	13	succinate dehydrogenase, flavoprotein subunit (sdhA)
NMB0951	13	succinate dehydrogenase, iron-sulfur protein (sdhB)
NMB0952	13	conserved hypothetical protein
NMB0953	8	hypothetical protein
NMB0954	12	citrate synthase (gltA)
NMB0956	13	2-oxoglutarate dehydrogenase, E2 component, dihydrolipoamide succinyltransferase (sucB)
NMB0957	13	2-oxoglutarate dehydrogenase, E3 component, lipoamide dehydrogenase (lpd)
NMB0958	13	hypothetical protein

NMB0959	13	succinyl-CoA synthetase, beta subunit (sucC)
NMB0960	10	succinyl-CoA synthetase, alpha subunit (sucD)
NMB0962	10	excinuclease ABC, subunit A (uvrA)
NMB0963	13	phosphatidylserine decarboxylase precursor-related protein
NMB0964	11	TonB-dependent receptor
NMB0965	12	hypothetical protein
NMB0966	12	para-aminobenzoate synthase glutamine amidotransferase component II (pabA)
NMB0967	13	anthranilate phosphoribosyltransferase (trpD)
NMB0969	9	IS1016C2 transposase
NMB0973	6	putative integral membrane protein
NMB0977	10	modulator of drug activity B (mdaB), putative
NMB0978	13	NAD(P) transhydrogenase, beta subunit (pntB)
NMB0979	12	hypothetical protein
NMB0980	10	NAD(P) transhydrogenase, alpha subunit (pntA)
NMB0981	13	phosphoserine phosphatase (serB)
NMB0982	7	chloride channel protein-related protein
NMB0983	6	phosphoribosylaminoimidazolecarboxamide formyltransferase-IMP cyclohydrolase (purH)
NMB0984	8	transposase
NMB0985	9	E16-related protein
NMB0986	6	hypothetical protein
NMB0987	6	N-acetylmuramoyl-L-alanine amidase, putative
NMB0988	7	hypothetical protein
NMB0989	9	hypothetical protein
NMB0990	11	hypothetical protein
NMB0991	13	IS1106 transposase
NMB0992	13	adhesin
NMB0993	13	rubredoxin
NMB0994	11	acyl-CoA dehydrogenase family protein
NMB0995	12	macrophage infectivity potentiator-related protein
NMB0996	9	hypothetical protein
NMB0997	11	D-lactate dehydrogenase (dld)
NMB0998	11	oxidoreductase, putative
NMB0999	8	NifR3/SMM1 family protein
NMB1000	13	IS1106 transposase
NMB1002	12	hypothetical protein
NMB1003	9	hypothetical protein
NMB1004	3	hypothetical protein
NMB1005	3	hypothetical protein
NMB1009	5	conserved hypothetical protein
NMB1012	11	hypothetical protein
NMB1013	12	hypothetical protein
NMB1016	12	conserved hypothetical protein
NMB1017	7	sulfate ABC transporter, periplasmic sulfate-binding protein (sbp)
NMB1018	8	conserved hypothetical protein
NMB1019	13	phosphoribosylaminoimidazole carboxylase, ATPase subunit (purK)
NMB1020	13	hypothetical protein
NMB1021	4	anthranilate synthase component I (trpE)
NMB1023	13	conserved hypothetical protein
NMB1024	13	conserved hypothetical protein
NMB1025	3	conserved hypothetical protein
NMB1026	13	conserved hypothetical protein
NMB1027	11	dnaJ protein (dnaJ)
NMB1028	12	conserved hypothetical protein
NMB1029	13	aspartate ammonia-lyase (aspA)
NMB1030	13	conserved hypothetical protein
NMB1031	13	3-isopropylmalate dehydrogenase (leuB)
NMB1032	12	type II restriction enzyme NlaIV (nlaIVR)
NMB1034	13	3-isopropylmalate dehydratase, small subunit (leuD)
NMB1035	6	hypothetical protein

NMB1036	3	3-isopropylmalate dehydratase, large subunit (leuC)
NMB1037	13	glutamate--cysteine ligase (gshA)
NMB1038	7	DNA repair protein RadC (radC)
NMB1039	7	conserved hypothetical protein
NMB1040	13	hypothetical protein
NMB1041	9	GTP-binding protein
NMB1042	4	cation transport ATPase, E1-E2 family
NMB1043	13	hypothetical protein
NMB1045	10	hypothetical protein
NMB1046	13	threonine synthase (thrC)
NMB1047	7	hypothetical protein
NMB1048	6	hypothetical protein
NMB1051	11	ABC transporter, ATP-binding protein
NMB1052	3	dedA protein (dedA)
NMB1054	13	IS1106 transposase
NMB1055	8	serine hydroxymethyltransferase (glyA)
NMB1059	13	conserved hypothetical protein
NMB1060	13	fructose-1,6-bisphosphatase (fbp)
NMB1061	12	conserved hypothetical protein
NMB1062	12	conserved hypothetical protein
NMB1063	13	dihydroneopterin aldolase (folB)
NMB1064	13	conserved hypothetical protein
NMB1065	13	crcB protein (crcB)
NMB1066	13	hypothetical protein
NMB1068	13	gamma-glutamyl phosphate reductase (proA)
NMB1069	10	glutamate 5-kinase (proB)
NMB1070	13	2-isopropylmalate synthase (leuA)
NMB1071	13	conserved hypothetical protein
NMB1072	13	prolipoprotein diacylglyceryl transferase (lgt)
NMB1073	9	conserved hypothetical protein
NMB1074	13	acetylglutamate kinase (argB)
NMB1075	13	conserved hypothetical protein
NMB1076	13	DnaA-related protein
NMB1077	12	ABC transporter with multiple termination codons
NMB1078	10	transcriptional regulator, UmuD/LexA family
NMB1079	3	hypothetical protein
NMB1080	3	ner protein
NMB1081	13	bacteriophage transposase
NMB1082	5	hypothetical protein
NMB1083	13	bacteriophage DNA transposition protein B, putative
NMB1084	3	hypothetical protein
NMB1085	6	N-acetylmuramoyl-L-alanine amidase, putative
NMB1087	5	hypothetical protein
NMB1089	11	hypothetical protein
NMB1090	11	hypothetical protein
NMB1091	11	hypothetical protein
NMB1092	6	hypothetical protein
NMB1093	4	hypothetical protein
NMB1094	5	hypothetical protein
NMB1095	5	conserved hypothetical protein
NMB1096	5	conserved hypothetical protein
NMB1097	3	cryptic Mu-phage G protein, putative
NMB1098	3	I protein, putative
NMB1100	3	hypothetical protein
NMB1102	4	hypothetical protein
NMB1103	4	hypothetical protein
NMB1104	3	phage sheath protein
NMB1105	3	hypothetical protein
NMB1110	5	tail protein, 43 kDa

NMB1111	6	baseplate assembly protein V, putative
NMB1112	4	conserved hypothetical protein
NMB1113	4	conserved hypothetical protein
NMB1114	3	conserved hypothetical protein
NMB1115	13	phage tail fibre gene
NMB1117	8	hypothetical protein
NMB1118	2	conserved hypothetical protein
NMB1119	6	conserved hypothetical protein
NMB1120	4	hypothetical protein
NMB1122	12	ABC transporter, ATP-binding protein
NMB1124	13	hypothetical protein
NMB1125	10	hypothetical protein
NMB1126	12	hypothetical protein
NMB1127	9	oxidoreductase, short chain dehydrogenase/reductase family
NMB1128	4	conserved hypothetical protein
NMB1129	2	hypothetical protein
NMB1130	13	phytoene synthase, putative
NMB1131	13	chaperone protein HscA (hscA)
NMB1132	13	hypothetical protein
NMB1133	12	conserved hypothetical protein - ankyrin-related protein
NMB1134	12	ferredoxin, 2Fe-2S type (fdx)
NMB1136	12	hypothetical protein
NMB1138	9	conserved hypothetical protein
NMB1139	13	acetyl-CoA carboxylase, carboxyl transferase alpha subunit (accA)
NMB1140	13	mesJ protein (mesJ)
NMB1141	13	RNA methyltransferase, TrmH family
NMB1142	13	hypothetical protein
NMB1144	13	hypothetical protein
NMB1145	9	UDP-N-acetylmuramate:L-alanyl-gamma-D-glutamyl-meso-diaminopimelate ligase (mpl)
NMB1146	10	biotin synthetase (bioB)
NMB1147	13	hypothetical protein
NMB1148	13	hypothetical protein
NMB1149	13	hypothetical protein
NMB1150	13	dihydroxy-acid dehydratase (ilvD)
NMB1151	9	sulfite reductase hemoprotein, beta-component (cysI)
NMB1152	9	sulfite reductase (NADPH) flavoprotein, alpha component (cysJ)
NMB1153	7	sulfate adenylyltransferase, subunit 1
NMB1154	3	sulfate adenylyltransferase, subunit 2
NMB1155	13	phosphoadenosine phosphosulfate reductase
NMB1156	9	fusion product between siroheme synthase and sulphate adenylyltransferase subunit 1
NMB1157	10	hypothetical protein
NMB1158	13	nickel-dependent hydrogenase, b-type cytochrome subunit
NMB1159	11	conserved hypothetical protein
NMB1161	13	hypothetical protein
NMB1162	13	hypothetical protein
NMB1163	10	hypothetical protein
NMB1164	12	hypothetical protein
NMB1165	9	oxidoreductase, short chain dehydrogenase/reductase family
NMB1166	4	conserved hypothetical protein
NMB1167	3	hypothetical protein
NMB1168	13	phytoene synthase, putative
NMB1169	13	chaperone protein HscA (hscA)
NMB1170	13	hypothetical protein
NMB1171	12	conserved hypothetical protein - ankyrin-related protein
NMB1172	12	ferredoxin, 2Fe-2S type (fdx)
NMB1174	12	hypothetical protein
NMB1176	9	conserved hypothetical protein
NMB1177	13	acetyl-CoA carboxylase, carboxyl transferase alpha subunit (accA)
NMB1178	13	mesJ protein (mesJ)

NMB1179	13	RNA methyltransferase, TrmH family
NMB1180	13	hypothetical protein
NMB1182	13	hypothetical protein
NMB1183	9	UDP-N-acetylmuramate:L-alanyl-gamma-D-glutamyl-meso-diaminopimelate ligase (mpl)
NMB1184	10	biotin synthetase (bioB)
NMB1185	13	hypothetical protein
NMB1186	13	hypothetical protein
NMB1187	13	hypothetical protein
NMB1188	13	dihydroxy-acid dehydratase (ilvD)
NMB1189	9	sulfite reductase hemoprotein, beta-component (cysI)
NMB1190	9	sulfite reductase (NADPH) flavoprotein, alpha component (cysJ)
NMB1191	7	sulfate adenylyltransferase, subunit 1
NMB1192	3	sulfate adenylyltransferase, subunit 2
NMB1193	13	phosphoadenosine phosphosulfate reductase
NMB1194	9	fusion product between siroheme synthase and sulphate adenylyltransferase subunit 1
NMB1195	10	hypothetical protein
NMB1196	13	nickel-dependent hydrogenase, b-type cytochrome subunit
NMB1197	11	conserved hypothetical protein
NMB1199	12	GTP-binding protein TypA (typA)
NMB1200	13	ribonuclease II family protein (vacB)
NMB1201	11	IMP dehydrogenase (guaB)
NMB1202	12	hypothetical protein
NMB1203	6	protein-PII uridylyltransferase (glnD)
NMB1204	8	putative transcriptional regulator
NMB1205	13	hypothetical protein
NMB1206	10	bacterioferritin B (bfrB)
NMB1207	13	bacterioferritin A (bfrA)
NMB1208	13	hypothetical protein
NMB1209	8	hypothetical protein
NMB1210	9	toxin-activating protein, putative
NMB1211	13	hypothetical protein
NMB1212	1	hypothetical protein
NMB1213	11	lipoprotein, putative
NMB1214	13	hemagglutinin/hemolysin-related protein
NMB1215	12	hypothetical protein
NMB1216	13	lipoic acid synthetase (lipA)
NMB1217	7	lipoate-protein ligase B (lipB)
NMB1218	10	conserved hypothetical protein
NMB1219	13	transporter, putative
NMB1220	10	stomatin/Mec-2 family protein
NMB1221	7	hypothetical protein
NMB1222	9	uracil-DNA glycosylase (ung)
NMB1225	9	hypothetical protein
NMB1226	7	ABC transporter, ATP-binding protein
NMB1228	10	homoserine dehydrogenase (metM)
NMB1229	13	hypothetical protein
NMB1230	11	DNA-binding protein HU-beta (hupB)
NMB1232	12	conserved hypthetical protein
NMB1233	9	exodeoxyribonuclease V, alpha subunit (recD)
NMB1234	7	ABC transporter, ATP-binding protein
NMB1234	7	ABC transporter, ATP-binding protein
NMB1235	13	conserved hypothetical protein
NMB1236	13	hypothetical protein
NMB1237	7	recombination protein RecR (recR)
NMB1238	13	peptidyl-prolyl cis-trans isomerase-related protein
NMB1239	13	conserved hypothetical protein
NMB1240	10	ABC transporter, ATP-binding protein
NMB1241	8	tRNA nucleotidyltransferase (cca)
NMB1242	10	hypothetical protein

NMB1243	8	Holliday junction DNA helicase RuvB (ruvB)
NMB1244	11	ribulose-phosphate 3-epimerase (rpe)
NMB1245	11	hypothetical protein
NMB1246	13	conserved hypothetical protein
NMB1247	13	riboflavin synthase, alpha subunit (ribE)
NMB1248	7	molybdopterin-guanine dinucleotide biosynthesis protein A (mobA)
NMB1249	12	nitrate-nitrite sensory protein NarX, putative
NMB1250	13	transcriptional regulator, LuxR family
NMB1252	5	phosphoribosylformylglycinamidine cyclo-ligase (purM)
NMB1253	13	hypothetical protein
NMB1254	13	GTP cyclohydrolase II (ribA)
NMB1255	11	glycosyl transferase, degenerate
NMB1256	7	GTP cyclohydrolase II-3,4-dihydroxy-2-butanone-4-phosphate synthase (ribA-B)
NMB1257	13	site-specific DNA methylase, degenerate
NMB1258	13	hypothetical protein
NMB1260	13	type III restriction-modification system EcoPI enzyme, subunit res (res)
NMB1261	13	Type III restriction system methylase
NMB1262	10	peptidyl-prolyl cis-trans isomerase
NMB1263	13	CobW-related protein
NMB1266	12	zinc uptake regulation protein, putative (zur)
NMB1267	13	low molecular weight protein tyrosine-phosphatase
NMB1269	10	hypothetical protein
NMB1270	13	conserved hypothetical protein
NMB1271	13	mercury transport periplasmic protein, putative
NMB1272	13	hypothetical protein
NMB1273	12	alginate O-acetylation protein AlgI, putative
NMB1274	12	hypothetical protein
NMB1275	10	hypothetical protein
NMB1275	6	hypothetical protein
NMB1276	7	long-chain-fatty-acid--CoA ligase (fadD-1)
NMB1277	13	transporter, BCCT family
NMB1278	10	site-specific recombinase (gcr)
NMB1279	13	membrane-bound lytic murein transglycosylase B, putative
NMB1280	13	very long chain acyl-CoA dehydrogenase-related protein
NMB1281	9	transcription-repair coupling factor (mfd)
NMB1282	13	aspartate 1-decarboxylase (panD)
NMB1283	6	2-dehydro-3-deoxyphosphooctonate aldolase (kdsA)
NMB1284	13	conserved hypothetical
NMB1285	13	enolase (eno)
NMB1287	9	ferredoxin, putative
NMB1288	13	ribonucleoside-diphosphate reductase, beta subunit (nrdB)
NMB1291	13	ribonucleoside-diphosphate reductase, alpha subunit (nrdA)
NMB1292	13	hypothetical protein
NMB1293	13	hypothetical protein
NMB1294	9	1-acyl-sn-glycerol-3-phosphate acyltransferase (plsC)
NMB1295	13	formamidopyrimidine-DNA glycosylase (mutM)
NMB1296	7	hypothetical protein
NMB1297	3	membrane-bound lytic murein transglycosylase D
NMB1298	10	ribosomal small subunit pseudouridine synthase A (rsuA)
NMB1299	10	sodium dependent transporter
NMB1300	9	cytidylate kinase (cmk)
NMB1301	9	30S ribosomal protein S1 (rpsA)
NMB1302	4	integration host factor, beta subunit (himD)
NMB1303	13	transcriptional regulator, MerR family
NMB1304	11	alcohol dehydrogenase, class III (adhC)
NMB1305	8	esterase (esd)
NMB1306	6	conserved hypothetical protein
NMB1307	13	nucleoside diphosphate kinase (ndk)
NMB1308	8	conserved hypothetical protein

NMB1309	13	fimbrial biogenesis and twitching motility protein, putative
NMB1310	13	gcpE protein (gcpE)
NMB1312	12	ATP-dependent Clp protease, proteolytic subunit (clpP)
NMB1313	13	trigger factor (tig)
NMB1314	4	cell division protein FtsK (ftsK-2)
NMB1315	8	uracil permease (uraA)
NMB1316	9	hypothetical protein
NMB1317	6	hypothetical protein
NMB1318	13	CDP-diacylglycerol--serine O-phosphatidyltransferse (pssA)
NMB1319	7	conserved hypothetical protein
NMB1320	6	50S ribosomal protein L9 (rplI)
NMB1321	13	30S ribosomal protein S18 (rpsR)
NMB1322	13	primosomal replication protein n, putative
NMB1323	13	30S ribosomal protein S6 (rpsF)
NMB1324	12	thioredoxin reductase (trxB)
NMB1325	13	cation transport ATPase, E1-E2 family
NMB1326	10	excinuclease ABC, subunit C (uvrC)
NMB1327	1	conserved hypothetical protein
NMB1328	13	conserved hypothetical protein
NMB1331	13	excinuclease ABC, subunit B (uvrB)
NMB1332	13	carboxy-terminal peptidase (prc)
NMB1333	12	conserved hypothetical protein
NMB1334	5	hypothetical protein
NMB1335	13	creA protein (creA)
NMB1336	8	conserved hypothetical protein
NMB1337	8	conserved hypothetical protein
NMB1338	13	isomerase, putative
NMB1339	10	prolyl-tRNA synthetase (proS)
NMB1340	5	hypothetical protein
NMB1341	13	pyruvate dehydrogenase, E1 component (pdhA)
NMB1342	12	pyruvate dehydrogenase, E2 component, dihydrolipoamide acetyltransferase (pdhB)
NMB1344	10	pyruvate dehydrogenase, E3 component, lipoamide dehydrogenase (lpdA)
NMB1345	13	hypothetical protein
NMB1346	10	dead TonB-dependent receptor
NMB1347	13	extragenic suppressor protein SuhB (suhB)
NMB1348	13	RNA methylase, putative
NMB1349	13	hypothetical protein
NMB1351	13	fmu and fmv protein, putative
NMB1352	13	hypothetical protein
NMB1353	12	aldehyde dehydrogenase family protein
NMB1354	13	conserved hypothetical protein
NMB1355	12	glutamyl-tRNA (Gln) amidotransferase subunit C, putative (gatC)
NMB1356	13	Glu-tRNA(Gln) amidotransferase, subunit A (gatA)
NMB1357	13	conserved hypothetical protein
NMB1358	9	Glu-tRNA(Gln) amidotransferase, subunit B (gatB)
NMB1359	13	CDP-6-deoxy-delta-3,4-glucoseen reductase, putative
NMB1360	13	pyridoxamine 5-phosphate oxidase (pdxH)
NMB1361	13	conserved hypothetical protein
NMB1362	12	oxalate/formate antiporter, putative
NMB1363	12	exodeoxyribonuclease, large subunit (xseA)
NMB1364	12	NH(3)-dependent NAD\+ synthetase NadE, putative
NMB1365	12	conserved hypothetical protein
NMB1366	13	thioredoxin
NMB1367	12	conserved hypothetical protein
NMB1368	3	ATP-dependent RNA helicase, putative
NMB1369	12	hypothetical protein
NMB1370	12	hypothetical protein
NMB1371	12	acetylornithine aminotransferase (argD)
NMB1372	13	ATP-dependent Clp protease, ATP-binding subunit ClpX (clpX)

NMB1373	13	ribosome-binding factor A (rbfA)
NMB1374	12	tRNA pseudouridine synthase B (truB)
NMB1375	13	Restriction system methylase
NMB1376	11	Type III restriction - modification system enzyme
NMB1377	13	L-lactate dehydrogenase (lldD)
NMB1378	8	conserved hypothetical protein
NMB1380	13	nifU protein
NMB1381	12	HesB-YadR-YfhF family protein
NMB1382	12	conserved hypothetical protein
NMB1383	10	chaperone protein HscB (hscB)
NMB1384	13	DNA gyrase subunit A (gyrA)
NMB1385	13	IS1016 family transposase
NMB1387	10	hypothetical protein
NMB1388	12	glucose-6-phosphate isomerase (pgi-2)
NMB1389	13	RpiR/YebK/YfhH family protein / transcriptional regulator
NMB1390	13	glucokinase (glk)
NMB1391	11	oxidoreductase, Sol/DevB family
NMB1392	13	glucose-6-phosphate 1-dehydrogenase (zwf)
NMB1393	13	phosphogluconate dehydratase (edd)
NMB1394	7	4-hydroxy-2-oxoglutarate aldolase-2-deydro-3-deoxyphosphogluconate aldolase (eda)
NMB1395	13	alcohol dehydrogenase, zinc-containing
NMB1396	13	A-G-specific adenine glycosylase (mutY)
NMB1397	10	hypothetical protein
NMB1399	13	IS1106 transposase
NMB1401	13	IS1016 transposase
NMB1411	13	IS1016 transposase
NMB1412	10	FrpC operon protein
NMB1413	13	IS1016 family transposase
NMB1414	10	FrpC operon protein
NMB1416	12	aminopeptidase N (pepN)
NMB1417	13	conserved hypothetical protein
NMB1418	13	HtrB/MsbB family protein
NMB1419	13	crossover junction endodeoxyribonuclease RuvC (ruvC)
NMB1420	9	factor-for-inversion stimulation protein Fis, putative
NMB1421	11	nifR3 protein
NMB1422	12	ATP-dependent RNA helicase, putative
NMB1423	13	conserved hypothetical protein
NMB1425	12	lysyl-tRNA synthetase, heat inducible (lysU)
NMB1426	13	hypothetical protein
NMB1428	10	aminopeptidase, putative
NMB1430	6	transcription elongation factor GreA (greA)
NMB1431	8	hypothetical protein
NMB1432	6	3-phosphoshikimate 1-carboxyvinyltransferase (aroA)
NMB1433	9	conserved hypothetical protein
NMB1434	1	cardiolipin synthetase family protein
NMB1435	3	drug resistance translocase family protein
NMB1436	12	conserved hypothetical protein
NMB1437	13	conserved hypothetical protein
NMB1438	9	conserved hypothetical protein
NMB1439	6	phosphoribosylaminoimidazole carboxylase, catalytic subunit (purE)
NMB1440	10	hypothetical protein
NMB1441	9	O-methyltransferase, putative
NMB1442	7	mismatch repair protein MutL (mutL)
NMB1443	6	DNA polymerase III, subunits gamma and tau (dnaX)
NMB1444	13	conserved hypothetical protein
NMB1445	13	recA protein (recA)
NMB1446	12	3-dehydroquinate dehydratase (aroD)
NMB1447	1	ATP-dependent DNA helicase Rep (rep)
NMB1448	13	DNA-damage-inducible protein P (dinP)

NMB1449	13	TonB-dependent receptor, authentic point mutation
NMB1450	13	ferredoxin--NADP reductase (fpr-2)
NMB1451	13	DNA polymerase III, epsilon subunit (dnaQ-1)
NMB1452	13	conserved hypothetical protein
NMB1453	12	hypothetical protein
NMB1454	13	ferredoxin, 4Fe-4S bacterial type
NMB1455	13	hypothetical protein
NMB1456	12	hypothetical protein
NMB1457	13	transketolase (tktA)
NMB1458	12	fumarate hydratase, class II (fumC)
NMB1459	13	conserved hypothetical protein
NMB1460	12	single-strand binding protein (ssb)
NMB1461	8	probable MFS family transporter protein
NMB1462	12	transglycosylase, putative
NMB1465	13	opacity protein (opa)
NMB1466	13	conserved hypothetical protein
NMB1467	12	exopolyphosphatase (ppx)
NMB1468	13	hypothetical protein
NMB1469	2	hypothetical protein
NMB1470	13	hypothetical protein
NMB1471	13	tryptophanyl-tRNA synthetase (trpS)
NMB1472	11	clpB protein (clpB)
NMB1473	13	aminotransferase, class I
NMB1474	13	4-oxalocrotonate tautomerase, putative
NMB1476	13	glutamate dehydrogenase, NAD-specific (gluD)
NMB1477	13	hypothetical protein
NMB1478	12	phosphoglycolate phosphatase (gph)
NMB1479	9	regulatory protein RecX (recX)
NMB1480	5	hypothetical protein
NMB1482	13	acyl CoA thioester hydrolase family protein
NMB1483	13	lipoprotein NlpD, putative
NMB1484	13	stationary-phase survival protein SurE
NMB1485	13	conserved hypothetical protein
NMB1487	10	fimbrial assembly protein (fimB)
NMB1488	13	succinate-semialdehyde dehydrogenase (NADP\+) (gabD)
NMB1489	10	hypothetical protein
NMB1491	11	hypothetical protein
NMB1492	9	hypothetical protein
NMB1493	13	carbon starvation protein A (cstA)
NMB1494	13	conserved hypothetical protein
NMB1496	12	conserved hypothetical protein
NMB1497	12	TonB-dependent receptor
NMB1498	6	aspartokinase, alpha and beta subunits (lysC)
NMB1499	5	ribonuclease PH (rph)
NMB1500	8	conserved hypothetical protein
NMB1501	4	HesA/MoeB/ThiF family protein
NMB1502	13	hypothetical protein
NMB1503	13	hypothetical protein
NMB1504	12	conserved hypothetical protein
NMB1505	11	nicotinate phosphoribosyltransferase (pncB)
NMB1506	10	arginyl-tRNA synthetase (argS)
NMB1507	9	hypothetical protein
NMB1508	8	hypothetical protein
NMB1509	6	amino acid ABC transporter, permease protein
NMB1510	2	thermonuclease family protein
NMB1511	13	ribose 5-phosphate isomerase A (rpiA)
NMB1512	8	YgbB-YacN family protein
NMB1513	1	conserved hypothetical protein
NMB1514	6	DNA polymerase III, epsilon subunit (dnaQ-2)

NMB1515	12	transporter, putative
NMB1516	12	fixS protein (fixS)
NMB1517	8	hypothetical protein
NMB1518	7	acetate kinase (ackA-2)
NMB1519	9	thiol:disulfide interchange protein DsbD (dsbD)
NMB1520	13	hypothetical protein
NMB1521	1	phytoene synthase-related protein
NMB1522	10	FKBP-type peptidyl-prolyl cis-trans isomerase SlyD (slyD)
NMB1523	2	Lip / H8 outer membrane protein
NMB1524	13	oxidoreductase, putative
NMB1526	13	small major protein B
NMB1527	6	ADP-heptose--LPS heptosyltransferase II (rfaF)
NMB1528	7	methylated-DNA--protein-cysteine methyltransferase, putative
NMB1529	8	dead hypothetical protein
NMB1530	8	succinyl-diaminopimelate desuccinylase (dapE)
NMB1531	9	conserved hypothetical protein
NMB1532	9	conserved hypothetical protein
NMB1533	12	H.8 outer membrane protein/ azurin like protein
NMB1534	13	hypothetical protein
NMB1535	13	hypothetical protein
NMB1536	4	preprotein translocase SecA subunit (secA)
NMB1537	6	DNA primase (dnaG)
NMB1538	6	RNA polymerase sigma factor RpoD (rpoD)
NMB1539	13	IS1106 transposase
NMB1540	7	lactoferrin-binding protein A (lbpA)
NMB1541	10	lactoferrin-binding protein B
NMB1549	5	hypothetical protein
NMB1553	9	IS1106 related transposase
NMB1554	13	CTP synthase (pyrG)
NMB1555	13	long-chain-fatty-acid--CoA ligase (fadD-2)
NMB1556	11	tRNA (5-methylaminomethyl-2-thiouridylate)-methyltransferase (trmU)
NMB1557	11	conserved hypothetical protein
NMB1558	10	diacylglycerol kinase (dgkA)
NMB1559	13	glutathione synthetase (gshB)
NMB1560	12	glutaminyl-tRNA synthetase (glnS)
NMB1561	13	transcriptional regulator, DeoR family
NMB1562	9	conserved hypothetical protein
NMB1563	9	transcriptional regulator, GntR family
NMB1564	12	conserved hypothetical protein
NMB1565	12	hypothetical protein
NMB1566	13	phosphoribosylglycinamide formyltransferase (purN)
NMB1567	13	macrophage infectivity potentiator
NMB1568	13	DNA polymerase holoenzyme chi subunit, putative
NMB1569	12	aminopeptidase A-I (pepA)
NMB1570	12	conserved hypothetical protein
NMB1571	10	conserved hypothetical protein
NMB1572	13	aconitate hydratase 2 (acnB)
NMB1573	10	ornithine carbamoyltransferase, catabolic (argF)
NMB1574	13	ketol-acid reductoisomerase (ilvC)
NMB1575	12	conserved hypothetical protein
NMB1576	13	acetolactate synthase III, small subunit (ilvH)
NMB1577	11	acetolactate synthase III, large subunit (ilvI)
NMB1578	8	conserved hypothetical protein
NMB1579	11	ATP phosphoribosyltransferase (hisG)
NMB1580	13	hypothetical protein
NMB1581	4	histidinol dehydrogenase (hisD)
NMB1582	12	histidinol-phosphate aminotransferase (hisC)
NMB1583	13	imidazoleglycerol-phosphate dehydratase (hisB)
NMB1584	9	3-hydroxyacid dehydrogenase

NMB1587	13	protease, putative
NMB1588	12	CDP-diacylglycerol--glycerol-3-phosphate 3-phosphatidyltransferase (pgsA)
NMB1589	12	hypothetical protein
NMB1591	7	transcriptional regulator MtrA (mtrA)
NMB1592	12	hypothetical protein
NMB1593	13	conserved hypothetical protein
NMB1594	12	spermidine-putrescine ABC transporter, periplasmic spermidine-putrescine-binding protein (potD-3)
NMB1595	13	alanyl-tRNA synthetase (alaS)
NMB1600	3	hypothetical protein
NMB1601	13	IS1106 transposase
NMB1602	8	transposase
NMB1604	4	phosphoglycerate mutase (gpm)
NMB1605	2	topoisomerase IV subunit A (parC)
NMB1606	7	sensor histidine kinase
NMB1607	9	sigma-54 dependent response regulator
NMB1608	13	conserved hypothetical protein
NMB1609	10	trans-sulfuration enzyme family protein
NMB1610	13	hypothetical protein
NMB1611	8	hypothetical protein
NMB1612	10	amino acid ABC transporter, periplasmic amino acid-binding protein
NMB1613	9	fumarate hydratase, class I (fumB)
NMB1614	12	Trk system potassium uptake protein TrkA (trkA)
NMB1615	7	hypothetical protein
NMB1616	9	phosphomethylpyrimidine kinase (thiD)
NMB1617	7	tellurite resistance protein, putative
NMB1618	7	ribonuclease HI (rnhA)
NMB1619	7	conserved hypothetical protein
NMB1620	13	conserved hypothetical protein
NMB1622	12	nitric oxide reductase (norB)
NMB1623	10	major anaerobically induced outer membrane protein (pan1)
NMB1624	9	conserved hypothetical protein
NMB1628	4	tspB
NMB1636	13	opacity protein (opa)
NMB1637	12	conserved hypothetical protein
NMB1638	10	YhbX-YhjW-YijP-YjdB family protein
NMB1639	10	hypothetical protein
NMB1640	13	phosphoserine aminotransferase (serC)
NMB1641	13	conserved hypothetical protein
NMB1642	12	N utilization substance protein A (nusA)
NMB1643	13	translation initiation factor IF-2 (infB)
NMB1644	6	hypothetical protein
NMB1645	7	hypothetical protein
NMB1647	10	amino acid symporter, putative
NMB1648	7	conserved hypothetical protein
NMB1649	7	disulfide bond formation protein B (dsbB)
NMB1650	10	leucine-responsive regulatory protein (lrp)
NMB1651	13	alanine racemase (alr)
NMB1652	7	conserved hypothetical protein
NMB1653	13	conserved hypothetical protein
NMB1654	5	conserved hypothetical protein
NMB1655	13	adenine specific methylase, putative
NMB1657	13	putative DNA transport competence protein
NMB1658	13	DNA/pantothenate metabolism flavoprotein (dfp)
NMB1659	9	guanosine-3`,5`-bis(diphosphate) 3`-pyrophosphohydrolase (spoT)
NMB1660	13	DNA-directed RNA polymerase, omega subunit (rpoZ)
NMB1661	12	guanylate kinase (gmk)
NMB1662	13	adenine phosphoribosyltransferase (apt)
NMB1663	13	conserved hypothetical protein
NMB1664	13	protease, putative

NMB1665	13	conserved hypothetical protein
NMB1666	13	hypothetical protein
NMB1667	13	hypothetical protein
NMB1669	10	iron-starvation protein PigA
NMB1670	12	PqiA family protein
NMB1671	12	pqiB protein (pqiB)
NMB1672	13	conserved hypothetical protein
NMB1673	13	DNA-3-methyladenine glycosylase I, putative
NMB1674	13	GDSL lipase family protein
NMB1676	12	glycine dehydrogenase (decarboxylating) (gcvP)
NMB1677	12	cytochrome c5
NMB1678	12	aromatic-amino-acid aminotransferase (tyrB)
NMB1679	13	tRNA (uracil-5-)-methyltransferase (trmA)
NMB1680	9	chorismate synthase (aroC)
NMB1681	9	hypothetical protein
NMB1682	8	topoisomerase IV subunit B (parE)
NMB1683	7	MutT-nudix family protein
NMB1684	13	seryl-tRNA synthetase (serS)
NMB1685	10	D-lactate dehydrogenase (ldhA)
NMB1686	9	peptide chain release factor 1 (prfA)
NMB1687	5	conserved hypothetical protein
NMB1688	11	L-asparaginase I (ansA)
NMB1689	9	dedA protein, putative
NMB1690	6	phosphoglucomutase-phosphomannomutase family protein (mrsA)
NMB1691	11	dihydropteroate synthase (folP)
NMB1693	12	hypothetical protein
NMB1694	12	conserved hypothetical protein
NMB1695	12	hypothetical protein
NMB1696	10	acyl carrier protein
NMB1697	5	acyl carrier protein, putative
NMB1698	13	acyltransferase, putative
NMB1699	12	hypothetical protein
NMB1700	9	hypothetical protein
NMB1701	1	hypothetical protein
NMB1702	10	3-oxoacyl-(acyl-carrier-protein) reductase (fabG-1)
NMB1703	13	3-oxoacyl-(acyl-carrier-protein) synthase II (fabF-2)
NMB1704	6	beta-1,4-glucosyltransferase (lgtF)
NMB1705	3	alpha-1,2-N-acetylglucosamine transferase (rfaK)
NMB1706	13	hypothetical protein
NMB1707	11	sodium- and chloride-dependent transporter
NMB1708	7	hypothetical protein
NMB1709	9	thymidylate synthase (thyA)
NMB1710	13	glutamate dehydrogenase, NADP-specific (gdhA)
NMB1711	12	transcriptional regulator, GntR family
NMB1714	13	multidrug efflux pump channel protein (mtrE)
NMB1715	12	multiple transferable resistance system protein MtrD (mtrD)
NMB1716	3	membrane fusion protein (mtrC)
NMB1717	13	trancscriptional regulator MtrR (mtrR)
NMB1718	8	hypothetical protein
NMB1719	3	efflux pump component MtrF (mtrF)
NMB1720	9	exodeoxyribonuclease V 125 kD polypeptide (recC)
NMB1721	10	conserved hypothetical protein
NMB1722	12	cytochrome C555, truncation
NMB1723	13	cytochrome c oxidase, subunit III (fixP)
NMB1724	10	cytochrome c oxidase, subunit II (fixO)
NMB1725	13	cytochrome c oxidase, subunit I (fixN)
NMB1726	13	conserved hypothetical protein
NMB1727	13	conserved hypothetical protein
NMB1728	13	biopolymer transport protein ExbD (exbD)

NMB1729	11	biopolymer transport protein ExbB (exbB)
NMB1730	11	TonB protein (tonB)
NMB1731	3	conserved hypothetical protein
NMB1734	13	glutaredoxin (grx)
NMB1735	10	GTP pyrophosphokinase (relA)
NMB1739	11	hypothetical protein
NMB1744	6	putative integral membrane protein
NMB1747	4	TspB
NMB1750	6	pilin gene inverting protein PivNM-2
NMB1752	13	conserved hypothetical protein
NMB1753	13	VapD-related protein
NMB1754	7	cryptic plasmid protein A-related protein,
NMB1755	11	hypothetical protein
NMB1756	12	hypothetical protein
NMB1757	12	hypothetical protein
NMB1759	11	conserved hypothetical protein
NMB1760	8	conserved hypothetical protein
NMB1761	12	conserved hypothetical protein
NMB1762	13	hemolysin activation protein HecB, putative
NMB1763	13	toxin-activating protein, putative
NMB1764	7	hypothetical protein
NMB1766	12	hypothetical protein
NMB1767	5	hypothetical protein
NMB1768	5	hemagglutinin/hemolysin-related protein
NMB1769	9	IS1016C2 transposase
NMB1777	1	hypothetical protein
NMB1785	8	hypothetical protein
NMB1788	12	ATP-dependent DNA helicase RecG (recG)
NMB1789	10	protein-export protein SecB (secB)
NMB1790	9	glutaredoxin 3 (grxC)
NMB1791	12	cytoplasmic axial filament protein (cafA)
NMB1796	2	conserved hypothetical protein
NMB1797	4	penicillin-binding protein 3
NMB1798	13	IS1016 family transposase
NMB1799	10	S-adenosylmethionine synthetase (metK)
NMB1800	2	hypothetical protein
NMB1802	13	O-sialoglycoprotein endopeptidase (gcp)
NMB1803	10	cytochrome c-type biogenesis protein, putative
NMB1804	12	cytochrome c-type biogenesis protein, putative
NMB1805	1	cytochrome c4
NMB1806	13	conserved hypothetical protein
NMB1808	8	pilM protein (pilM)
NMB1810	9	pilO protein (PilO)
NMB1811	13	pilP protein (pilP)
NMB1812	10	pilQ protein (pilQ)
NMB1814	8	3-dehydroquinate synthase (aroB)
NMB1815	2	conserved hypothetical protein
NMB1816	6	conserved hypothetical protein
NMB1817	8	riboflavin-specific deaminase (ribD)
NMB1819	13	hypothetical protein
NMB1820	6	pilin glycosylation protein PglB (pglB)
NMB1821	6	pilin glycosylation protein PglC (pglC)
NMB1823	7	valine--pyruvate aminotransferase (avtA)
NMB1824	7	conserved hypothetical protein
NMB1825	13	hypothetical protein
NMB1826	2	conserved hypothetical protein
NMB1827	9	DNA polymerase III, alpha subunit (dnaE)
NMB1828	12	conserved hypothetical protein
NMB1829	11	TonB dependent receptor

NMB1830	2	phosphoglycolate phosphatase, putative
NMB1831	13	lytB protein (lytB)
NMB1832	11	lipoprotein signal peptidase (lspA)
NMB1833	9	isoleucyl-tRNA synthetase (ileS)
NMB1834	12	riboflavin kinase/FMN adenylyltransferase (ribF)
NMB1835	8	tyrosyl-tRNA synthetase (tyrS)
NMB1836	10	lipopolysaccharide biosynthesis protein WbpC, putative
NMB1837	13	hypothetical protein
NMB1838	13	GTP-binding protein, putative
NMB1839	13	formate--tetrahydrofolate ligase (fhs)
NMB1840	13	conserved hypothetical protein
NMB1841	13	mannose-1-phosphate guanyltransferase-related protein
NMB1842	13	4-hydroxyphenylacetate 3-hydroxylase, small subunit, putative
NMB1843	13	transcriptional regulator, MarR family
NMB1845	13	thioredoxin
NMB1846	12	Mrp-NBP35 family protein
NMB1848	13	hypothetical protein
NMB1849	13	carbamoyl-phosphate synthase, small subunit (carA)
NMB1850	11	hypothetical protein
NMB1851	12	hypothetical protein
NMB1852	10	conserved hypothetical protein
NMB1853	13	hypothetical protein
NMB1854	12	hypothetical protein
NMB1855	10	carbamoyl-phosphate synthase, large subunit (carB)
NMB1856	9	transcriptional regulator, LysR family
NMB1857	12	modulator of drug activity B (mdaB)
NMB1858	2	hypothetical protein
NMB1859	12	S-adenosylmethionine:tRNA ribosyltransferase-isomerase (queA)
NMB1860	13	acetyl-CoA carboxylase, biotin carboxyl carrier protein (accB)
NMB1861	12	acetyl-CoA carboxylase, biotin carboxylase (accC)
NMB1862	13	ribosomal protein L11 methyltransferase (prmA)
NMB1863	13	oligoribonuclease (orn)
NMB1864	7	glutamate-1-semialdehyde 2,1-aminomutase (hemL)
NMB1865	13	hypothetical protein
NMB1866	9	conserved hypothetical protein
NMB1867	12	1-deoxyxylulose-5-phosphate synthase (dxs)
NMB1868	6	integrase/recombinase XerC (xerC)
NMB1869	4	fructose-bisphosphate aldolase (cbbA)
NMB1871	4	conserved hypothetical protein
NMB1872	13	ribosomal-protein-alanine acetyltransferase, putative
NMB1873	3	DNA polymerase, bacteriophage-type, putative
NMB1874	1	orotate phosphoribosyltransferase (pyrE)
NMB1875	7	hypothetical protein
NMB1876	12	N-acetylglutamate synthase (argA)
NMB1877	8	prolyl oligopeptidase family protein
NMB1878	13	transcriptional regulator, AraC family
NMB1884	12	conserved hypothetical protein
NMB1885	13	protein-L-isoaspartate O-methyltransferase (pcm)
NMB1886	9	conserved hypothetical protein
NMB1887	13	triosephosphate isomerase (tpiA)
NMB1888	8	protein-export membrane protein SecG (secG)
NMB1892	7	hypothetical protein
NMB1893	13	conserved hypothetical protein
NMB1894	13	leucyl-tRNA synthetase, truncation
NMB1895	13	DNA adenine methylase, truncation
NMB1896	13	type II restriction enzyme DpnI (dpnC)
NMB1897	13	leucyl-tRNA synthetase (leuS)
NMB1898	13	lipoprotein (mlp)
NMB1899	7	hypothetical protein

NMB1900	12	polyphosphate kinase (ppk)
NMB1901	13	IS1016 transposase
NMB1902	13	DNA polymerase III, beta subunit (dnaN)
NMB1903	13	chromosomal replication initiator protein DnaA (dnaA)
NMB1904	13	ribosomal protein L34 (rpl34)
NMB1905	7	ribonuclease P protein component (rnpA)
NMB1906	8	conserved hypothetical protein
NMB1907	9	60 kd inner-membrane protein (yidC)
NMB1908	10	conserved hypothetical protein
NMB1909	13	Maf/YceF/YhdE family protein
NMB1910	9	conserved hypothetical protein
NMB1911	6	50S ribosomal protein L32 (rpmF)
NMB1912	13	conserved hypothetical protein
NMB1913	13	fatty acid-phospholipid synthesis protein (plsX)
NMB1914	13	hypothetical protein
NMB1915	13	hypothetical protein
NMB1916	13	3-oxoacyl-(acyl-carrier-protein) synthase III (fabH)
NMB1917	8	conserved hypothetical protein
NMB1918	13	malonyl CoA-acyl carrier protein transacylase (fabD)
NMB1919	12	ABC transporter, ATP-binding protein
NMB1920	13	GMP synthase (guaA)
NMB1921	13	3-oxoacyl-(acyl-carrier-protein) reductase (fabG-2)
NMB1923	12	conserved hypothetical protein
NMB1924	13	inositol monophosphatase family protein
NMB1925	13	conserved hypothetical protein
NMB1927	13	lacto-N-neotetraose biosynthesis glycosyl transferase-related protein
NMB1928	13	lacto-N-neotetraose biosynthesis glycosyl transferase LgtB (lgtB)
NMB1929	13	lacto-N-neotetraose biosynthesis glycosyl transferase LgtA (lgtA)
NMB1930	11	glycyl-tRNA synthetase, beta chain (glyS)
NMB1931	12	hypothetical protein
NMB1932	12	glycyl-tRNA synthetase, alpha chain (glyQ)
NMB1933	13	ATP synthase F1, epsilon subunit (atpC)
NMB1934	13	ATP synthase F1, beta subunit (atpD)
NMB1935	13	ATP synthase F1, gamma subunit (atpG)
NMB1936	13	ATP synthase F1, alpha subunit (atpA)
NMB1938	6	ATP synthase F0, B subunit (atpF)
NMB1939	13	ATP synthase F0, C subunit (atpE)
NMB1940	13	ATP synthase F0, A subunit (atpB)
NMB1941	13	hypothetical protein
NMB1944	12	ParB family protein
NMB1945	13	3-octaprenyl-4-hydroxybenzoate carboxy-lyase (ubiX)
NMB1946	13	outer membrane lipoprotein
NMB1947	13	ABC transporter, permease protein
NMB1948	12	ABC transporter, ATP-binding protein
NMB1949	13	soluble lytic murein transglycosylase, putative
NMB1950	12	30S ribosomal protein S21 (rpsU)
NMB1951	13	conserved hypothetical protein
NMB1952	12	stringent starvation protein B (sspB / regG)
NMB1953	13	stringent starvation protein A (sspA / regF)
NMB1954	13	hypothetical protein
NMB1955	13	cadmium resistance protein
NMB1956	13	50S ribosomal protein L31 (rpmE)
NMB1958	12	thioredoxin, putative
NMB1959	13	conserved hypothetical protein
NMB1960	13	hypothetical protein
NMB1961	12	VacJ-related protein
NMB1962	10	hypothetical protein
NMB1963	13	conserved hypothetical protein
NMB1964	12	conserved hypothetical protein

NMB1965	9	conserved hypothetical protein
NMB1966	11	ABC transporter, ATP-binding protein
NMB1967	13	transcriptional regulator, AraC family
NMB1968	12	aldehyde dehydrogenase A (aldA)
NMB1970	13	para-aminobenzoate synthetase component I-4-amino-4-deoxychorismate lyase, putative (pabB-C)
NMB1971	10	conserved hypothetical protein
NMB1972	13	chaperonin, 60 kDa (groEL)
NMB1973	2	chaperonin, 10 kDa (groES)
NMB1974	13	IS1016 transposase
NMB1975	12	sodium- and chloride-dependent transporter
NMB1976	10	diaminopimelate decarboxylase (lysA)
NMB1977	9	hypothetical protein
NMB1978	9	cyaY protein (cyaY)
NMB1979	11	conserved hypothetical protein
NMB1980	10	conserved hypothetical protein
NMB1981	9	conserved hypothetical protein
NMB1982	9	DNA polymerase I (polA)
NMB1983	13	hypothetical protein
NMB1984	13	IS1106 transposase
NMB1985	9	adhesion and penetration protein (hap)
NMB1986	13	hypothetical protein
NMB1987	9	thiophene and furan oxidation protein ThdF (thdF)
NMB1988	3	iron-regulated outer membrane protein FrpB (frpB)
NMB1989	13	iron(III) ABC transporter, periplasmic binding protein
NMB1990	13	iron(III) ABC transporter, permease protein
NMB1991	13	iron(III) ABC transporter, permease protein
NMB1992	3	hypothetical protein
NMB1993	13	iron(III) ABC transporter, ATP-binding protein
NMB1995	4	nitrogen regulatory protein P-II (glnB)
NMB1996	3	phosphoribosylformylglycinamidine synthase (purL)
NMB1997	4	hydroxyacylglutathione hydrolase (hagH)
NMB1999	13	magnesium transporter (mgtE)
NMB2001	3	conserved hypothetical protein
NMB2002	5	hypothetical protein
NMB2003	12	conserved hypothetical protein
NMB2004	10	conserved hypothetical protein
NMB2005	6	glutamate N-acetyltransferase/amino-acid acetyltransferase (argJ)
NMB2006	6	chloride channel protein-related protein
NMB2007	7	ATP-dependent RNA helicase HrpA, truncation
NMB2009	13	ATP-dependent RNA helicase HrpA, degenerate
NMB2010	4	YhbX/YhjW/YijP/YjdB family protein
NMB2011	6	ATP-dependent RNA helicase HrpA,
NMB2015	13	hypothetical protein
NMB2016	11	type IV pilin-related protein ComP (comP)
NMB2017	13	putative DNA transport competence protein
NMB2019	13	lipopolysaccharide core biosynthesis protein KdtB (kdtB)
NMB2020	12	conserved hypothetical protein
NMB2021	7	conserved hypothetical protein
NMB2022	6	conserved hypothetical protein
NMB2023	13	conserved hypothetical protein
NMB2024	12	conserved hypothetical protein
NMB2025	6	conserved hypothetical protein
NMB2026	4	ABC transporter, permease protein
NMB2029	7	homoserine kinase (thrB)
NMB2030	10	3-demethylubiquinone-9 3-methyltransferase (ubiG)
NMB2031	13	tryptophan transporter (mtr)
NMB2032	12	lipopolysaccharide glycosyl transferase, (lgtG)
NMB2033	1	histidinol-phosphatase, putative
NMB2034	12	1-acyl-sn-glycerol-3-phosphate acyltransferase, putative

NMB2035	6	conserved hypothetical protein
NMB2036	13	tRNA pseudouridine synthase A (truA)
NMB2039	12	major outer membrane protein PIB (porB)
NMB2040	13	thiamine biosynthesis protein ThiC (thiC)
NMB2041	13	thiamin pyrophosphokinase-related protein
NMB2042	13	spermidine-putrescine ABC transporter, ATP-binding protein (potA-2)
NMB2043	13	IS1106 transposase
NMB2044	13	phosphoenolpyruvate-protein phosphotransferase (ptsI)
NMB2045	13	phosphocarrier protein HPr (ptsH)
NMB2046	13	PTS system, IIAB component
NMB2047	13	hypoxanthine-guanine phosphoribosyltransferase, putative
NMB2048	13	DNA ligase (ligA-2)
NMB2049	11	glyoxalase II family protein
NMB2050	10	conserved hypothetical protein
NMB2051	13	ubiquinol--cytochrome c reductase, cytochrome c1 (petC)
NMB2052	13	ubiquinol--cytochrome c reductase, cytochrome b (petB)
NMB2053	11	ubiquinol--cytochrome c reductase, iron-sulfur subunit (petA)
NMB2054	13	conserved hypothetical protein
NMB2055	10	transcriptional regulator, LysR family
NMB2056	5	30S ribosomal protein S9 (rpsI)
NMB2057	12	50S ribosomal protein L13 (rplM)
NMB2058	11	conserved hypothetical protein
NMB2059	8	hypothetical protein
NMB2060	13	glycerol-3-phosphate dehydrogenase (NAD\+) (gpsA)
NMB2061	3	phosphoenolpyruvate carboxylase (ppc)
NMB2062	7	thiF protein (thiF)
NMB2063	13	slyX protein, putative
NMB2064	7	conserved hypothetical protein
NMB2065	7	hemK protein (hemK)
NMB2066	12	tldD protein (tldD)
NMB2067	6	conserved hypothetical protein
NMB2068	9	D-amino acid oxidase flavoprotein, putative
NMB2069	7	thiamin-phosphate pyrophosphorylase (thiE)
NMB2070	9	hypothetical protein
NMB2071	9	thiG protein (thiG)
NMB2072	7	hypothetical protein
NMB2073	9	hypothetical protein
NMB2074	13	hypothetical protein
NMB2075	7	BirA protein/Bvg accessory factor
NMB2076	10	aut protein (aut)
NMB2077	7	methylenetetrahydrofolate dehydrogenase-methenyltetrahydrofolate cyclohydrolase (folD)
NMB2078	9	conserved hypothetical protein
NMB2079	7	aspartate-semialdehyde dehydrogenase (asd)
NMB2080	3	hypothetical protein
NMB2081	6	hypothetical protein
NMB2082	8	exodeoxyribonuclease (exoA)
NMB2083	7	cysteinyl-tRNA synthetase (cysS)
NMB2085	13	hypothetical protein
NMB2086	13	GTP-binding protein
NMB2088	12	conserved hypothetical protein
NMB2089	13	hypothetical protein
NMB2090	13	phosphoheptose isomerase (gmhA)
NMB2091	13	hemolysin, putative
NMB2092	13	hypothetical protein
NMB2093	10	methionine aminopeptidase (map)
NMB2094	8	hypothetical protein
NMB2095	13	adhesin complex protein, putative
NMB2096	10	malate:quinone oxidoreductase (yojH)
NMB2097	7	hypothetical protein

NMB2098	13	conserved hypothetical protein
NMB2099	10	conserved hypothetical protein
NMB2100	13	hypothetical protein
NMB2101	11	30S ribosomal protein S2 (rpsB)
NMB2102	12	elongation factor TS (EF-TS) (tsf)
NMB2103	12	uridylate kinase (pyrH)
NMB2104	11	MafA protein sequence
NMB2105	10	mafB protein (mafB)
NMB2106	13	hypothetical protein
NMB2107	13	alternative C-terminus for mafB
NMB2109	13	hypothetical protein
NMB2110	13	hypothetical protein
NMB2111	13	alternative C-terminus for mafB
NMB2112	13	hypothetical protein
NMB2113	13	hypothetical protein
NMB2114	13	alternative C-terminus for mafB
NMB2115	12	hypothetical protein
NMB2116	13	hypothetical protein
NMB2117	13	alternative C-terminus for mafB
NMB2118	13	hypothetical protein
NMB2119	13	alternative C-terminus for mafB
NMB2120	13	hypothetical protein
NMB2121	13	hypothetical protein
NMB2123	13	hypothetical protein
NMB2125	13	hypothetical protein
NMB2126	13	IS1016 family transposase
NMB2127	8	protease, putative
NMB2128	12	CinA-related protein
NMB2129	12	argininosuccinate synthase (argG)
NMB2130	12	hypothetical protein
NMB2132	12	transferrin-binding protein-related protein
NMB2133	13	sodium/dicarboxylate symporter family protein
NMB2134	12	conserved hypothetical protein
NMB2135	13	conserved hypothetical protein
NMB2136	12	peptide transporter
NMB2137	12	hypothetical protein
NMB2138	11	peptide chain release factor 2 (prfB)
NMB2139	12	conserved hypothetical protein
NMB2140	13	conserved hypothetical protein
NMB2141	9	hypothetical protein
NMB2142	13	conserved hypothetical protein
NMB2143	13	conserved hypothetical protein
NMB2144	5	sigma factor, putative
NMB2145	13	hypothetical protein
NMB2146	7	hypothetical protein
NMB2147	13	hypothetical protein
NMB2150	13	conserved hypothetical protein
NMB2151	13	phosphoribosylamine--glycine ligase (purD)
NMB2152	11	hypothetical protein
NMB2153	13	conserved hypothetical protein
NMB2154	9	electron transfer flavoprotein, alpha subunit (etfA)
NMB2155	13	electron transfer flavoprotein, beta subunit (etfB)
NMB2156	13	heptosyltransferase I (rfaC)
NMB2157	11	pyrazinamidase/nicotinamidase PncA, putative
NMB2158	6	conserved hypothetical protein
NMB2159	4	glyceraldehyde 3-phosphate dehydrogenase (gapA-2)
NMB2160	5	DNA mismatch repair protein MutS (mutS)
NMA0001	5	hypothetical protein
NMA0024	13	hypothetical protein

NMA0036	10	hypothetical protein
NMA0038	13	hypothetical protein
NMA0039	13	hypothetical protein
NMA0040	12	hypothetical protein
NMA0041	6	hypothetical protein
NMA0046	7	putative transmembrane transport protein
NMA0047	4	hypothetical protein
NMA0051	9	hypothetical protein
NMA0053	11	hypothetical protein
NMA0068	13	hypothetical protein
NMA0089	13	hypothetical protein
NMA0131	13	hypothetical protein
NMA0132A	13	hypothetical protein
NMA0140	4	hypothetical protein
NMA0170	12	putative lipoprotein
NMA0171A	8	hypothetical protein
NMA0179	12	hypothetical protein
NMA0212	9	hypothetical protein
NMA0223	10	hypothetical protein
NMA0236	13	hypothetical protein
NMA0318	13	hypothetical protein
NMA0322	13	hypothetical protein
NMA0324	13	mafB protein (mafB)
NMA0330	13	conserved hypothetical protein
NMA0360	12	hypothetical protein
NMA0366	13	hypothetical protein
NMA0372	13	very hypothetical protein
NMA0395	13	hypothetical protein
NMA0396	7	hypothetical protein
NMA0398	12	major outer membrane protein PIB (porB)
NMA0426A	7	hypothetical protein
NMA0432	6	hypothetical protein
NMA0455	12	hypothetical protein
NMA0474	9	hemoglobin-haptoglobin untilization protein (hpuB)
NMA0475	8	hemoglobin-haptoglobin utilization protin (hpuA)
NMA0481	12	hypothetical protein
NMA0482	13	hypothetical protein
NMA0500	13	hypothetical protein
NMA0527	13	lacto-N-neotetraose biosynthesis glycosyl tranferase
NMA0598	11	hypothetical protein
NMA0606	13	hypothetical protein
NMA0629	12	hypothetical protein
NMA0630	10	hypothetical protein
NMA0631	13	hypothetical protein
NMA0640	13	hypothetical protein
NMA0641	7	conserved hypothetical protein
NMA0677	6	hypothetical protein
NMA0686	8	putative ABC transporter protein
NMA0701	1	hypothetical protein
NMA0772	6	putative DNA-invertase
NMA0773	6	hypothetical protein
NMA0774	6	hypothetical protein
NMA0775	6	possible integral membrane protein
NMA0778	6	hypothetical protein
NMA0779	4	putative integral membrane protein
NMA0780	6	hypothetical protein
NMA0781	6	hypothetical protein
NMA0782	3	putative phage replication protein
NMA0783	6	putative phage replication protein

NMA0787	3	hypothetical protein
NMA0799	8	hypothetical protein
NMA0814	13	hypothetical protein
NMA0817	13	putative polyamine permease inner membrane protein
NMA0845	13	hypothetical protein
NMA0854	12	hypothetical protein
NMA0855	12	hypothetical protein
NMA0856	12	hypothetical protein
NMA0857	13	hypothetical protein
NMA0858	5	hypothetical protein
NMA0860	3	bis(5 prime nucleosyl)-tetraphosphatase (apaH)
NMA0871	12	hypothetical protein
NMA0877	13	hypothetical protein
NMA0883	8	putative lipoprotein
NMA0936	1	hypothetical protein
NMA0939	13	hypothetical protein
NMA0951	13	conserved hypothetical
NMA0971	4	putative lipoprotein
NMA0973	10	putative integral membrane protein
NMA1014	1	hypothetical protein
NMA1075	8	hypothetical protein
NMA1081	4	putative membrane protein
NMA1082	13	hypothetical protein
NMA1083	2	putative periplasmic protein
NMA1131	13	hypothetical protein
NMA1157	6	hypothetical protein
NMA1158	12	hypothetical protein
NMA1165	7	hypothetical protein
NMA1173	2	putative cell-surface protein
NMA1174	2	hypothetical protein
NMA1190	13	hypothetical protein
NMA1194	7	hypothetical protein
NMA1195	13	hypothetical protein
NMA1208	7	hypothetical protein
NMA1211	13	hypothetical protein
NMA1229	13	putative membrane protein
NMA1231	13	hypothetical protein
NMA1258	13	conserved hypothetical protein
NMA1282	13	putative DNA-binding protein
NMA1283	13	hypothetical protein
NMA1293	13	putative phage anti-repressor protein
NMA1294	13	hypothetical protein
NMA1305	13	hypothetical protein
NMA1308	10	hypothetical protein
NMA1311	2	hypothetical protein
NMA1312	8	putative DNA-binding protein
NMA1313	4	hypothetical protein
NMA1326	4	hypothetical protein
NMA1426	12	hypothetical protein
NMA1435	13	hypothetical protein
NMA1436	11	hypothetical protein
NMA1442	6	ferredoxin--NADP reductase (fpr-1)
NMA1484	12	hypothetical protein
NMA1489	11	hypothetical protein
NMA1587	13	hypothetical protein
NMA1595	9	hypothetical protein
NMA1602	10	hypothetical protein
NMA1615	10	hypothetical protein
NMA1637	13	hypothetical protein

NMA1654	13	hypothetical protein
NMA1690	13	hypothetical protein
NMA1692	13	putative membrane peptidase
NMA1697	7	hypothetical protein
NMA1723	13	Lip / H8 outer membrane protein
NMA1733	3	H.8 outer membrane protein
NMA1752	10	hypothetical protein
NMA1780	13	hypothetical protein
NMA1789	2	hypothetical protein
NMA1791	9	hypothetical protein
NMA1807	9	hypothetical protein
NMA1827	1	hypothetical protein
NMA1828	3	hypothetical protein
NMA1829	5	hypothetical protein
NMA1830	4	hypothetical protein
NMA1831	3	hypothetical protein
NMA1832	1	hypothetical protein
NMA1833	4	hypothetical DNA-binding protein
NMA1838	6	hypothetical DNA-binding protein
NMA1840	3	hypothetical protein
NMA1841	3	hypothetical protein
NMA1842	2	hypothetical protein
NMA1843	6	hypothetical protein
NMA1847	2	conserved hypothetical protein
NMA1848	4	hypothetical protein
NMA1849	5	hypothetical protein
NMA1850	3	hypothetical protein
NMA1851	4	conserved hypothetical protein
NMA1852	2	hypothetical protein
NMA1858	7	hypothetical protein
NMA1862	13	hypothetical protein
NMA1866	4	putative integral membrane protein
NMA1871	6	hypothetical protein
NMA1872	11	hypothetical protein
NMA1873	13	hypothetical protein
NMA1874	10	hypothetical protein
NMA1881	5	hypothetical DNA-binding protein
NMA1884	4	putative regulator
NMA1904	13	hypothetical protein
NMA1924	9	hypothetical protein
NMA1954	12	hypothetical protein
NMA1978	13	hypothetical protein
NMA2029	2	hypothetical protein
NMA2087	12	putative transcriptional regulator
NMA2116	13	hypothetical protein
NMA2120	13	hypothetical protein
NMA2121	12	hypothetical protein
NMA2137	13	hypothetical protein
NMA2149	9	putative inner membrane hypothetical protein
NMA2172	4	conserved hypothetical protein
NMA2222	10	hypothetical protein
NMA2230	5	hypothetical protein
XNG0034	13	hypothetical protein
XNG0062	11	Opa protein
XNG0065	11	Opa protein
XNG0080	13	hypothetical protein
XNG0081	13	conserved hypothetical protein
XNG0116	4	hypothetical protein
XNG0117	3	hypothetical protein

XNG0118	5	dead colicin secretion type ATP transporter
XNG0143	3	conserved hypothetical protein
XNG0150	8	hypothetical protein
XNG0178	9	spermidine/putrescine ABC transporter, permease protein (potC)
XNG0189	13	glycosyltransferase
XNG0194	10	phosphoribosylformimino-5-aminoimidazole carboxamide ribotide isomerase (hisA)
XNG0200	13	hypothetical protein
XNG0210	12	hypothetical protein
XNG0211	11	hypothetical protein
XNG0212	12	hypothetical protein
XNG0213	11	hypothetical protein
XNG0214	12	hypothetical protein
XNG0217	2	ribonuclease, putative
XNG0234	7	acetyl-CoA carboxylase, carboxyl transferase beta subunit (accD)
XNG0255	9	hypothetical protein
XNG0256	7	hypothetical protein
XNG0270	12	conserved hypothetical protein
XNG0272	9	RNA polymerase sigma-32 factor (rpoH)
XNG0284	13	very short patch repair endonuclease
XNG0285	12	restriction system methylase
XNG0304	13	hypothetical protein
XNG0311	7	conserved hypothetical protein
XNG0322	11	hypothetical protein
XNG0360	13	hypothetical protein
XNG0369	13	conserved hypothetical protein
XNG0375	13	transcriptional regulator, TetR family
XNG0387	4	DNA damage inducible protein D
XNG0388	11	hypothetical protein
XNG0388	7	hypothetical protein
XNG0418	7	pantoate--beta-alanine ligase (panC)
XNG0419	11	conserved hypothetical protein
XNG0433	13	fimT homologue
XNG0438	11	hypothetical protein
XNG0446	2	hypothetical protein
XNG0453	5	hypothetical protein
XNG0454	4	hypothetical protein
XNG0457	1	hypothetical protein
XNG0468	5	hypothetical protein
XNG0470	5	bacteriophage endodoxyribonuclease
XNG0471	2	hypothetical protein
XNG0472	2	hypothetical protein
XNG0473	1	hypothetical protein
XNG0475	8	hypothetical protein
XNG0494	8	hypothetical protein
XNG0497	5	hypothetical protein
XNG0498	2	hypothetical protein
XNG0501	2	hypothetical protein
XNG0503a	11	hypothetical protein
XNG0511	13	sugar efflux transporter
XNG0550	13	hypothetical protein
XNG0562	13	hypothetical protein
XNG0571	12	conserved hypothetical protein
XNG0606	5	cell filamentation protein
XNG0607	5	hypothetical protein
XNG0610	13	L-lactate dehydrogenase (lldD)
XNG0612	13	Restriction system methylase
XNG0636	11	hypothetical protein
XNG0662	13	hypothetical protein
XNG0664	9	conserved hypothetical protein

XNG0665	8	Type I restriction enzyme R protein
XNG0666	13	hypothetical protein
XNG0667	12	Type I restriction modification system specificity (S) protein
XNG0668	1	hypothetical protein
XNG0670	13	Type I restriction enzyme M protein
XNG0672	11	hemagglutinin/hemolysin related protein
XNG0674	12	hypothetical protein
XNG0688	3	hypothetical protein
XNG0690	3	baseplate assembly protein V from bacteriophage P2
XNG0697	5	hypothetical protein
XNG0703	11	DNA polymerase III, subunits gamma and tau (dnaX)
XNG0705	13	hypothetical protein
XNG0711	13	hypothetical protein
XNG0714	6	molybdopterin-guanine dinucleotide biosynthesis protein A
XNG0716	13	conserved hypothetical protein
XNG0726	13	hypothetical protein
XNG0731	3	pilin gene inverting protein PivNM-1B (pivNM-1B)
XNG0732	11	conserved hypthetical protein
XNG0763	13	hypothetical protein
XNG0810	4	surface protein with limited homology with opc
XNG0828	7	hypothetical protein
XNG0841	9	plasmid stability protein stbB
XNG0842	11	plasmid stability protein
XNG0861	13	50S ribosomal protein L31, putative (rpmE)
XNG0871	12	conserved hypothetical protein
XNG0877	11	Opa protein
XNG0910	13	transcriptional regulator
XNG0920	6	hypothetical protein
XNG0921	13	hypothetical protein
XNG0923	6	hypothetical protein
XNG0924	12	hypothetical protein
XNG0925	4	hypothetical protein
XNG0927	4	conserved hypothietical
XNG0928	7	hypothetical protein
XNG0930	11	hypothetical protein
XNG0931	11	hypothetical protein
XNG0932	11	hypothetical protein
XNG0933	11	hypothetical protein
XNG0938	8	hypothetical protein
XNG0952	11	Opa protein
XNG0954	12	exopolyphosphatase (ppx)
XNG0983	13	alternative C-terminus for mafB
XNG0987	11	Opa protein
XNG0993	12	hypothetical protein
XNG0994	6	hypothetical protein
XNG0995	5	hypothetical protein
XNG0996	7	conserved hypothetical protein
XNG1003	6	hypothetical protein
XNG1004	1	hypothetical protein
XNG1005	3	hypothetical protein
XNG1006	3	hypothetical protein
XNG1011	3	hypothetical protein
XNG1012	3	hypothetical protein
XNG1013	5	hypothetical protein
XNG1014	5	bacteriophage endodoxyribonuclease
XNG1024	6	hypothetical protein
XNG1025	1	hypothetical protein
XNG1027	4	hypothetical protein
XNG1028	5	hypothetical protein

XNG1038	11	hypothetical protein
XNG1041	13	putative glycosyl transferase
XNG1042	3	pilin gene inverting protein PivNM-1B (pivNM-1B)
XNG1043	4	hypothetical protein
XNG1053	6	hypothetical protein
XNG1061	7	hypothetical protein
XNG1062	13	hypothetical protein
XNG1064	13	hypothetical protein
XNG1068	3	pilin gene inverting protein PivNM-1B (pivNM-1B)
XNG1080	5	hypothetical protein
XNG1082	7	comEA-related protein
XNG1084a	5	16S ribosomal RNA sequence
XNG1105	3	pilin gene inverting protein PivNM-1B (pivNM-1B)
XNG1110	13	hypothetical protein
XNG1114	13	hypothetical protein
XNG1115	8	cytosine specific restriction modification system methylase
XNG1116	13	conserved hypothetical protein
XNG1163	10	sensor histidine kinase
XNG1165	3	pilin gene inverting protein PivNM-1B (pivNM-1B)
XNG1181	11	Opa protein
XNG1184	13	hypothetical protein
XNG1200	13	conserved hypothetical protein
XNG1205	5	hypothetical protein
XNG1207	7	comEA-related protein
XNG1207a	5	16S ribosomal RNA sequence
XNG1209	13	guanosine-3`,5`-bis(diphosphate) 3`-pyrophosphohydrolase (spoT)
XNG1229	10	tRNA (uracil-5-)-methyltransferase (trmA)
XNG1254	10	thiamine biosynthesis lipoprotein ApbE (apbE)
XNG1284	6	hypothetical protein
XNG1286	8	hypothetical protein
XNG1287	8	hypothetical protein
XNG1288	13	hypothetical protein
XNG1289	12	hypothetical protein
XNG1290	7	hypothetical protein
XNG1297	13	membrane protein (nosY)
XNG1300	13	nitrous oxide reductase (nosZ)
XNG1327	13	hypothetical protein
XNG1328	11	hypothetical protein
XNG1329	13	hypothetical protein
XNG1330	12	hypothetical protein
XNG1331	13	ABC transporter
XNG1332	8	cadmium resistance protein
XNG1335	13	thiol:disulfide interchange protein DsbC (dsbC)
XNG1341	13	surface protein, adhesin
XNG1352	11	Opa protein
XNG1380	11	transferrin-binding protein 2 (tbp2)
XNG1391	3	DNA repair protein RecO (recO)
XNG1396	11	Opa protein
XNG1400	12	hypothetical protein
XNG1437	11	Opa protein
XNG1441	9	hypothetical protein
XNG1460	13	conserved hypothetical protein
XNG1465	13	hypothetical protein
XNG1471	8	hypothetical protein
XNG1491	3	hypothetical protein
XNG1498	5	hypothetical protein
XNG1499	4	hypothetical protein
XNG1500	1	hypothetical protein
XNG1504	5	hypothetical protein

XNG1505	13	hypothetical protein
XNG1509	7	hypothetical protein
XNG1510	5	hypothetical protein
XNG1511	13	hypothetical protein
XNG1513	5	bacteriophage endodoxyribonuclease
XNG1514	2	hypothetical protein
XNG1515	3	pilin gene inverting protein PivNM-1B (pivNM-1B)
XNG1523	2	hypothetical protein
XNG1524	2	hypothetical protein
XNG1532	9	conserved hypothetical protein
XNG1569	4	hypothetical protein
XNG1573	7	comEA-related protein
XNG1576	5	16S ribosomal RNA sequence
XNG1582	9	hypothetical protein
XNG1583	3	pilin gene inverting protein PivNM-1B (pivNM-1B)
XNG1585	10	conserved hypothetical protein
XNG1644	8	glycosyltransferase (pglA)
XNG1648	13	cytochrome C551 peroxidase precursor (EC 1.11.1.5)
XNG1661	12	hypothetical protein
XNG1675	4	Type II restriction enzyme (similar to NgoPII)
XNG1691	12	hypothetical protein
XNG1723	13	50S ribosomal protein L23 (rplW)
XNG1731	6	hypothetical protein
XNG1733	11	hypothetical protein
XNG1733	10	hypothetical protein
XNG1739	12	50S ribosomal protein L1 (rplA)
XNG1745	13	conserved hypothetical protein
XNG1746	11	Opa protein
XNG1779	12	glucose-1-phosphate thymidylyltransferase (rfbA-1)
XNG1784	7	hypothetical protein
XNG1785	7	comEA-related protein
XNG1788	5	16S ribosomal RNA sequence
XNG1820	7	conserved hypothetical protein
XNG1855	12	hypothetical protein
XNG1856	12	hypothetical protein
XNG1868	13	methionine aminopeptidase (map)
XNG1872	13	conserved hypothetical protein
XNG1888	9	hypothetical protein
XNG1895	10	amino acid ABC transporter, permease protein
XNG1896	7	amino acid ABC transporter, integral membrane protein
XNG1897	7	amino acid ABC transporter, ATP-binding protein
XNG1898	12	amino acid transporter, periplasmic solute-binding protein
XNG1904	5	glycerol-3-phosphate dehydrogenase (NAD\+) (gpsA)
XNG1909	4	30S ribosomal protein S9 (rpsI)
XNG1910	8	hypothetical protein
XNG1948	11	Opa protein
XNG1968	12	lipopolysaccharide glycosyl transferase, (lgtG)
XNG1983	13	hypothetical protein
XNG1993	1	hypothetical protein
XNG2005	6	conserved hypothetical protein
XNG2007	13	hypothetical protein
XNG2014	13	hypothetical protein
XNG2045	13	lacto-N-neotetraose biosynthesis glycosyl transferase LgtA (lgtA)
XNG2047	13	galactose alpha 1-4 transferase LgtC (lgtC)
XNG2048	13	GalNAc transferase to lacto-N-neotetraose LgtD (lgtD)
XNG2052	13	conserved hypothetical protein
XNG2059	13	hypothetical protein
NMC0006	13	putative glycerate dehydrogenase
NMC0026	3	hypothetical protein

NMC0209	6	hypothetical protein
NMC0282	3	hypothetical protein
NMC0295	9	putative methylase
NMC0296B	9	hypothetical transposase interupted gene
NMC0298	9	hypothetical protein
NMC0300	7	hypothetical protein
NMC0393	10	hypothetical protein
NMC0399B	5	hypothetical protein
NMC0487	13	putative pilin
NMC0590	13	hypothetical protein
NMC0778	11	hypothetical protein
NMC0874	7	hypothetical protein
NMC0887	1	hypothetical protein
NMC0968	1	hypothetical protein
NMC1051	9	hypothetical protein
NMC1107	9	bacterioferritin B (bfrB)
NMC1120a	7	hypothetical protein
NMC1127	3	hypothetical protein
NMC1195	12	peptidyl-prolyl cis-trans isomerase (ppiA)
NMC1225	13	hypothetical protein
NMC1402	7	hypothetical protein
NMC1413	13	putative glutamate dehydrogenase
NMC1519	10	hypothetical protein
NMC1520	9	hypothetical protein
NMC1690	7	transferrin-binding protein A (tbpA)
NMC1691	9	transferrin-binding protein B (tbpB)
NMC1704	13	DNA repair protein RecO (recO)
NMC1795	13	hypothetical protein
NMC1812	8	putative ABC transporter, ATP-binding protein ( )
NMC1881	9	hypothetical protein
NMC2020	12	major outer membrane protein PorB (porB)
NMC2022	13	hypothetical protein
NMC2036	7	putative transcriptional activator protein MetR (metR)
NMC2141	13	glutamyl-tRNA synthetase (gltX)
GB CDS	10	Type IV pilin class II (pilE) (U81551)
GB CDS	13	truncated protein disrupted by IS element (AJ278708)
GB CDS	4	haloacid dehalogenase-like hydrolase, putative (AF320320)
GB CDS	12	pilin glycosylation protein PglB2 (AF320320)
GB CDS	2	putative methylase (AJ238948)
GB CDS	13	exl3L (AF319537)
GB CDS	13	MNgoNXV (AJ004687)
GB CDS	1	plasmid repressor protein (AF126482)
MME CDS	13	CDS1 from MME*frr* in *N. lactamica* (DQ115758)
MME CDS	13	CDS2 from MME*frr* in *N.lactamica* (DQ115758)
MME CDS	13	CDS from MME*nrdAB* in *N. lactamica* (DQ115765)
MME CDS	13	ORF1a from MME*pheST* in *N. polysaccharea* (AF542178)
MME CDS	13	ORF4 from MME*pheST* in *N. lactamica* (AF542177)
MME CDS	12	ORF1 from MME*pheST* in *N. lactamica* (AF542177)
MME CDS	12	CDS2 from MME*uvrB* in *N. meningitidis* strain 00/240794 (DQ115766)
MME CDS	11	CDS from MME*hesA* in *N. polysaccharea* (DQ115773)
NG5043	7	*yea* from *N. gonorrhoeae* strain MS11 Gonococcal Genetic Island (AY803022)
NG5020	6	*traN* from *N. gonorrhoeae* strain MS11 Gonococcal Genetic Island (AY803022)
NG5006	6	*traA* from *N. gonorrhoeae* strain MS11 Gonococcal Genetic Island (AY803022)
NG5053	1	*topB* from *N. gonorrhoeae* strain MS11 Gonococcal Genetic Island (AY803022)
cassette	3	ampicillin resistance gene
cassette	3	erythromycin resistance gene
16S_BIG	13	16S ribosomal RNA sequence
16S_MID	13	16S ribosomal RNA sequence
16S_SM	13	16S ribosomal RNA sequence

23S_BIG	13	23S ribosomal RNA sequence
23S_MID	13	23S ribosomal RNA sequence
23S_SM	13	23S ribosomal RNA sequence

[a]**Gene:** Indicates the gene probe that is hybridized by one or more of the *N. lactamica* strains tested. Genes are first identified by their locus annotation numbers from *N. meningitidis* serogroup B strain MC58[9]. When the gene probe is not present in the genome sequence of *N. meningitidis* serogroup B strain MC58, then the gene locus identifiers are from, in order, the *N. meningitidis* serogroup A strain Z2491 annotation[10], our *N. gonorrhoeae* strain FA1090 annotation (http://www.compbio.ox.ac.uk/data), *Neisseria* spp. sequences from GenBank/EMBL (Accession numbers listed in annotation column), sequences identified from Minimal Mobile Elements (Accession numbers listed in annotation column), sequences from the *N. gonorrhoeae* strain MS11 Gonococcal Genetic Island (AY803022), sequences from antibiotic resistance cassettes, and sequences from the rRNA loci.

[b]**Present:** Indicates the number of *N. lactamica* strains, out of 13 strains tested, which had strong hybridization signal for the indicated gene. Strong signal was determined by a pON value of > 0.67, as determined by BlueFuse for Microarrays (BlueGnome).

[c]**Annotation:** Derived from the published annotations and our own annotation of *N. gonorrhoeae* strain FA1090 and other sequences. This annotation of N. gonorrhoeae is displayed in the supporting FA1090 GBrowse database (http://www.compbio.ox.ac.uk/data).
