# Supplementary material for: The majority of genes in the pathogenic Neisseria species are present in non-pathogenic Neisseria lactamica, including those designated as 'virulence genes'
Source: BMC Genomics. 2006 May 30;7:128. doi: 10.1186/1471-2164-7-128 (PMC1538595; doi:10.1186/1471-2164-7-128)
Supplement: Additional file 3 — Probe content and design for the pan-Neisseria microarray, versions 1 & 2. This file provides a detailed description of the design and construction of the pan-Neisseria microarray and the revised and updated pan-Neisseria microarray-v2. Included are details of the probe design and selection criteria, the sequences against which the microarray was designed, additions to the microarray in version 2, and publications that have used the pan-Neisseria microarray in CGH. [file 1471-2164-7-128-S3.pdf]

Probe content and design for the pan-*Neisseria* microarray, versions 1 & 2.

For the pan-*Neisseria* microarray-v1, each of the then published genome sequences were compared using AceDB [1] as described previously [2, 3] using: *N. gonorrhoeae* strain FA1090 (AE004969), *N. meningitidis* strain MC58 [4], and *N. meningitidis* strain Z2491 [5]. At the time, the gonococcal genome sequence had not yet been annotated, therefore the first step in the design of the microarray was to annotate the *N. gonorrhoeae* strain FA1090 genome sequence. This was done using the methods and tools described for the annotation of *N. meningitidis* strain MC58 by the Oxford annotation team [4]. This annotation is available in an interactive comparative database, which contains our annotation, and those that have been released from the genome sequencing projects (http://www.compbio.ox.ac.uk/data).

For each annotated coding region within *N. gonorrhoeae* strain FA1090, a region was selected for probe design based on the homology of that region with the equivalent region within the orthologous genes from each of the two meningococcal genome sequences, while avoiding low-complexity regions. In this way, the gonococcal genome sequence-based probes were selected to target the most conserved sections of each gene in all three genomes as far as possible. These selected regions were then processed for probe and primer design for the PCR product probes using Primer3. Probes were designed that were 150 to 450 bp, unless the coding region was smaller than 150 bp. It has been independently demonstrated [6] that this is an ideal probe length for reproducible, good quality hybridization.

The region to be amplified by the Primer3 selected primers for each probe designed in this first round were added to the three AceDBs for assessment against the genome sequences. Probes were not approved until they met two criteria. Firstly, the probe must have ≥ 90% sequence identity over at least 150 bp of the probe for all instances of the gene. For *N. gonorrhoeae* strain FA1090, the probes for the gene against which they were designed were naturally 100% identical. For the meningococcal genome sequences, if the gonococcal sequence designed probe had < 90% sequence identity over 150 bp, and a more similar specific region could not be identified, then a region was selected from the meningococcal sequence so that an additional probe could be designed using the meningococcus as a template. Secondly, the specificity of the probe was determined. No probe was accepted if there was sequence identity of ≥ 80 % over 20 bp. For paralogous genes (homologues encoding different proteins present in the same genome sequence) separate probes were sought if possible, but if the homology of these sequences was such that separate probes could not be obtained to sufficiently divergent regions, then it was noted that this probe was for multiple genes. However, some regions within closely related genes have sufficient sequence divergence to allow unique and non-cross-hybridizing probes to be designed, for example for the related genes *pilC*1 and *pilC*2 in both species.

Having completed the first round of probe design, accepting those probes that have sufficient identity and rejecting those with low specificity, a second round of design was performed. In this round, any probes that were rejected were redesigned to avoid the identified potentially cross-hybridizing regions. In addition, those genes within the *N. meningitidis* strain MC58 genome sequence that did not have probes, due to their absence from the gonococcal genome sequence, or for which the gonococcal probes were sub-optimal, were used to design additional probes. These second round probes

were assessed and triaged similarly, and a third round of design was conducted adding in the unique genes from *N. meningitidis* strain Z2491 that did not have probes, or did not have optimal probes.

Probes were also designed against the then incomplete *N. gonorrhoeae* strain MS11 Gonococcal Genetic Island sequence [7], although these could not be designed with the same strategy due to the lack of availability of additional sequences of this 'island'. Two, further rounds of triage and design followed by manual primer selection completed the probe set.

The pan-*Neisseria* microarray-v1 contains 2704 probes to annotated coding regions and 6 different probes to rRNA sequences (3 for 16S and 3 for 23S). These probes cover 6294 of the 6408 annotated CDSs in the three genome sequences, including 75 repeated copies of transposases, for which only 6 probes were needed. The 114 annotated elements without probes are those that were determined not to be coding sequences, gene fragments, annotated intergenic sequences, silent gene cassettes, and transposase fragments. Therefore, 99.98% of the genome sequence features considered to have the potential to encode proteins have probes on the microarray. The average hybridization probe length is 230 base pairs. The average identity between the targeted sequence and its primary probe is 99.4%. The average target sequence identity of the primary probes for each gene is 99.7% for *N. gonorrhoeae* strain FA1090, 96.9% for *N. meningitidis* strain MC58, and 96.6% for *N. meningitidis* strain Z2491.

The pan-*Neisseria* microarray-v1 has been used previously in one CGH publication, which compared the gene complements of *N. gonorrhoeae* strains FA1090, FA19, F62, and MS11 [8].

With the completion of the *N. meningitidis* strain FAM18 genome sequence (Sanger Institute, unpublished, ftp://ftp.sanger.ac.uk/pub/pathogens/nm/) and the completion of the Gonococcal Genetic Island sequence [7], the design for the pan-*Neisseria* microarray-v2 was begun, using the same strategy as for the first version of the microarray. Some of the probes from the v1 microarray did not meet the $\geq$ 90% identity threshold over at least 150 bp for orthologues in *N. meningitidis* strain FAM18. New probes were designed for these. It was later discovered that hybridization of the v1 probes worked as well as the FAM18-specific v2 probes in the hybridization conditions used, although the v1 probes were as little as 83% identical over 150 bp. In addition to these sequences, the sequences of genes that were present in the public EMBL and GenBank databases, but were not present in any of the four neisserial genome sequences for which templates could be obtained were also included, as were new genes from our on-going investigations of neisserial Minimal Mobile Elements [9, 10]. In two instances of very short annotated coding sequences it was not possible to obtain a PCR product (genes: NMB0953 and XNG1580), and for these an oligonucleotide probe representing the whole of the coding sequence was generated and used as a probe on the microarray.

The pan-*Neisseria* microarray-v2 contains probes for the genes from the genome sequences of *N. gonorrhoeae* strain FA1090, *N. meningitidis* strain MC58, *N. meningitidis* strain Z2491, *N. meningitidis* strain FAM18, and to the genes from the *N. gonorrhoeae* strain MS11 Gonococcal Genetic Island, neisserial genes from

GenBank/EMBL that did not yet have probes, and newly identified genes from Minimal Mobile Elements (see Supplementary Table 2). These 2845 probes make this the most comprehensive microarray that could have been designed at the time and it has been specifically designed for use in comparative studies of expression changes in different strains, and for the CGH assessment of the gene complements of collections of neisserial strains. The positions and sequences of these probes can be accessed through the genome browser at http://www.compbio.ox.ac.uk/data, and a complete Array Definition File for this microarray has been submitted to MIAMExpress, accessible from the EBI (http://www.ebi.ac.uk/arrayexpress/) under the Array design name: BPFG PanNeisseriaArrray v2 (although this is not applicable for submission of bacterial CGH studies as reported in this paper). DNA templates used in the generation of the PCR product probes were: *N. gonorrhoeae* strain FA1090 for all 'X' probes; *N. meningitidis* strain MC58 for all 'B' probes; *N. meningitidis* strain Z2491 for all 'A' probes; *N. meningitidis* strain FAM18 for all 'C' probes; and as otherwise indicated in the probe description for all other probes. No false positive hybridizations have been noted when control hybridizations are conducted with the genome sequence strains under CGH the conditions used in this study.

This is the second publication using the pan-*Neisseria* microarray-v2; the first used CGH to investigate the presence of the GGI in *N. meningitidis* strains [10].

The pan-*Neisseria* microarray-v1 is available from Prof. John Davies, Monash University, Australia (e-mail: John.Davies@med.monash.edu.au). The pan-*Neisseria* microarray-v2 is available on a collaborative basis from Dr. Nigel Saunders, University of Oxford, UK (e-mail: Nigel.Saunders@path.ox.ac.uk).

References:

1. Durbin R, Thierry-Mieg JT: **A *C. elegans* DataBase. Documentation, code and data available from http://www.acedb.org**.
2. Saunders NJ, Jeffries AC, Peden JF, Hood DW, Tettelin H, Rappuoli R, Moxon ER: **Repeat-associated phase variable genes in the complete genome sequence of *Neisseria meningitidis* strain MC58.** *Molecular Microbiology* 2000, **37**(1):207-215.
3. Snyder LA, Butcher SA, Saunders NJ: **Comparative whole-genome analyses reveal over 100 putative phase-variable genes in the pathogenic *Neisseria* spp.** *Microbiology (Reading, England)* 2001, **147**(Pt 8):2321-2332.
4. Tettelin H, Saunders NJ, Heidelberg J, Jeffries AC, Nelson KE, Eisen JA, Ketchum KA, Hood DW, Peden JF, Dodson RJ *et al*: **Complete genome sequence of *Neisseria meningitidis* serogroup B strain MC58.** *Science* 2000, **287**(5459):1809-1815.
5. Parkhill J, Achtman M, James KD, Bentley SD, Churcher C, Klee SR, Morelli G, Basham D, Brown D, Chillingworth T *et al*: **Complete DNA sequence of a serogroup A strain of *Neisseria meningitidis* Z2491.** *Nature* 2000, **404**(6777):502-506.
6. Chou CC, Chen CH, Lee TT, Peck K: **Optimization of probe length and the number of probes per gene for optimal microarray analysis of gene expression.** *Nucleic Acids Res* 2004, **32**(12):e99.
7. Hamilton HL, Dominguez NM, Schwartz KJ, Hackett KT, Dillard JP: ***Neisseria gonorrhoeae* secretes chromosomal DNA via a novel type IV secretion system.** *Mol Microbiol* 2005, **55**(6):1704-1721.

8. Snyder LA, Davies JK, Saunders NJ: **Microarray genomotyping of key experimental strains of *Neisseria gonorrhoeae* reveals gene complement diversity and five new neisserial genes associated with Minimal Mobile Elements.** *BMC Genomics* 2004, **5**(1):23.
9. Saunders NJ, Snyder LAS: **The minimal mobile element.** *Microbiology* 2002, **148**(Pt 12):3756-3760.
10. Snyder LAS, Jarvis SA, Saunders NJ: **Complete and variant forms of the 'Gonococcal Genetic Island' in *Neisseria meningitidis*.** *Microbiology* 2005, **151**(Pt 12):4005-4013.
