# Supplementary material for: The majority of genes in the pathogenic Neisseria species are present in non-pathogenic Neisseria lactamica, including those designated as 'virulence genes'
Source: BMC Genomics. 2006 May 30;7:128. doi: 10.1186/1471-2164-7-128 (PMC1538595; doi:10.1186/1471-2164-7-128)
Supplement: Additional file 1 — Virulence-associated and predicted virulence-associated genes from the pathogenic Neisseria spp. This file contains a table of those genes from the current published literature that have been suggested to have a role in virulence. Genes for which there were positive hybridizations on the pan-Neisseria microarray-v2 in the N. lactamica strains tested are indicated, as are those genes present in all four of the currently available Neisseria spp. genome sequences. [file 1471-2164-7-128-S1.pdf]

Supplementary Table 1. Virulence-associated and predicted virulence-associated genes from the pathogenic *Neisseria* spp.[a]

ABC transport (NMB1880) *
**adhesin (NMB0586) ***
***bcp* ***
***bfrA* ***
***bfrB* ***
ctrA
ctrB
ctrC
ctrD
dca / *pptA* *
***dsbA* ***
**endonuclease (NMB0533) ***
***envA* ***
***fabZ* ***
***fbpA* ***
***fbpB* ***
frpA
frpA-related (NMB1407) *
frpC *
***fur* ***
***hap* ***
***hecB* (hemolysin activation)**
**hemagglutinin/hemolysin (NMB0493)**
**hemagglutinin/hemolysin (NMB1214)**
**hemagglutinin/hemolysin (NMB1768)**
hemaglutinin/hemolysin (NMB0497 & NMB1779)
***hemH* ***
hemolysin (NMB1646) *
hemolysin (NMB1738)
hemolysin activator (NMB0496 & NMB1780)
hmbR *
hsf
IgA protease *
**iron ABC transport system (NMB1989) ***
**iron ABC transport system (NMB1990) ***
**iron ABC transport system (NMB1991) ***
***kat* ***
***kdtA* ***
***lbpA* ***
lbpB
***lgtA* ***
***lgtB* ***
lgtC
lgtD
lgtE
***lgtF* ***

lgtG
lipA
lipB
***lpxA* ***
***lpxB* ***
***lpxD* ***
**macrophage infectivity potentiator (NMB0995) ***
**macrophage infectivity potentiator (NMB1567) ***
***mtrR* ***
***mtrC* ***
***mtrD* ***
***mtrE* ***
nadA
narE
***nlpD* ***
NMB0065
***norZ* ***
***omp*85 ***
***ompH* ***
***opa* ***
opc
**outer membrane lipoprotein (NMB1946) ***
***pglA* / *pgtA* ***
***pglB* ***
***pglC* ***
pglD *
***pglE* ***
pglF *
pglG
pglH
***pglI* / *wbpC* ***
***pgm* ***
***phoP / misR* ***
***phoQ / misS* ***
pilC *
pilD *
***pilE* ***
***pilF* ***
***pilG* ***
***pilT* ***
pilV *
porA *
***porB* ***
***prc* (peptidase) ***
**protease (NMB2127) ***
***rfaC* ***
***rfaD* ***
***rfaE* ***

***rfaF* ***
***rfaK* ***
***rmpM* ***
sacA
sacB
sacC
sacD
secretion protein (NMB1737)
siaA
siaB
siaC
siaD (serogroup B & C alleles)
**sialyltransferase (NMB0922) ***
***sodB* ***
sodC
*ssa*1 *
***tbp*1 (*tbpA*)**
***tbp*2 (*tbpB*)**
TonB-dependent receptor (NMB0293)
**TonB-dependent receptor (NMB1346) ***
**TonB-dependent receptor (NMB1449)**
**TonB-dependent receptor (NMB1829)**
TonB-dependent receptor (NMB1882) *
**toxin-activating (NMB1210)**
**toxin-activating (NMB1763)**
**transferrin binding protein (NMB2132) ***
***tspA* ***
***tspB* ***
**twitching motility protein (NMB0051) ***
***vacJ* ***
***vapA* ***
**VapD-related (NMB1753)**
virG *

[a]Genes listed in bold face are those present in at least one of the strains of *N. lactamica* tested in this study.

*Genes that are present in all of the four pathogenic *Neisseria* spp. genome sequences.
